# In-Depth Matrisome and Glycoproteomic Analysis of Human Brain Glioblastoma *Versus* Control Tissue

**DOI:** 10.1016/j.mcpro.2022.100216

**Published:** 2022-02-23

**Authors:** Manveen K. Sethi, Margaret Downs, Chun Shao, William E. Hackett, Joanna J. Phillips, Joseph Zaia

**Affiliations:** 1 Department of Biochemistry, Center for Biomedical Mass Spectrometry, Boston University, Boston, Massachusetts, USA; 2 Bioinformatics Program, Boston University, Boston, Massachusetts, USA; 3 Department of Neurological Surgery, Brain Tumor Center, Helen Diller Family Cancer Research Center, University of California San Francisco, San Francisco, California, USA; 4 Division of Neuropathology, Department of Pathology, University of California San Francisco, San Francisco, California, USA

**Keywords:** glioblastoma, matrisome, extracellular matrix, proteomics, glycomics, glycoproteins, glycosylation, mass spectrometry, glycosaminoglycans, proteoglycans, CLA, classical, CS, chondroitin sulfate, CSPGs, chondroitin sulfate proteoglycans, ECM, extracellular matrix, EGFR, epidermal growth factor receptor, GAG, glycosaminoglycan, GBM, glioblastoma, HPRO, hydroxyprolinated, HS, heparan sulfate, HSPGs, heparan sulfate proteoglycans, IDH1, Isocitrate dehydrogenase 1, LC-MS/MS, liquid chromatography-tandem mass spectrometry, MES, mesenchymal, PDGFRA, platelet-derived growth factor receptor alpha, PG, proteoglycan, PRO, proneural, RTK, receptor tyrosine kinase, sulf, sulfatase, TCGA, The Cancer Genome Atlas, TMA, tissue microarray

## Abstract

Glioblastoma (GBM) is the most common and malignant primary brain tumor. The extracellular matrix, also known as the matrisome, helps determine glioma invasion, adhesion, and growth. Little attention, however, has been paid to glycosylation of the extracellular matrix components that constitute the majority of glycosylated protein mass and presumed biological properties. To acquire a comprehensive understanding of the biological functions of the matrisome and its components, including proteoglycans (PGs) and glycosaminoglycans (GAGs), in GBM tumorigenesis, and to identify potential biomarker candidates, we studied the alterations of GAGs, including heparan sulfate (HS) and chondroitin sulfate (CS), the core proteins of PGs, and other glycosylated matrisomal proteins in GBM subtypes *versus* control human brain tissue samples. We scrutinized the proteomics data to acquire in-depth site-specific glycoproteomic profiles of the GBM subtypes that will assist in identifying specific glycosylation changes in GBM. We observed an increase in CS 6-*O* sulfation and a decrease in HS 6-*O* sulfation, accompanied by an increase in unsulfated CS and HS disaccharides in GBM *versus* control samples. Several core matrisome proteins, including PGs (decorin, biglycan, agrin, prolargin, glypican-1, and chondroitin sulfate proteoglycan 4), tenascin, fibronectin, hyaluronan link protein 1 and 2, laminins, and collagens, were differentially regulated in GBM *versus* controls. Interestingly, a higher degree of collagen hydroxyprolination was also observed for GBM *versus* controls. Further, two PGs, chondroitin sulfate proteoglycan 4 and agrin, were significantly lower, about 6-fold for isocitrate dehydrogenase-mutant, compared to the WT GBM samples. Differential regulation of *O*-glycopeptides for PGs, including brevican, neurocan, and versican, was observed for GBM subtypes *versus* controls. Moreover, an increase in levels of glycosyltransferase and glycosidase enzymes was observed for GBM when compared to control samples. We also report distinct protein, peptide, and glycopeptide features for GBM subtypes comparisons. Taken together, our study informs understanding of the alterations to key matrisomal molecules that occur during GBM development. (Data are available *via* ProteomeXchange with identifier PXD028931, and the peaks project file is available at Zenodo with DOI 10.5281/zenodo.5911810).

## Highlights

• Differential regulation of core matrisome proteins for GBM *versus* controls.
• A higher degree of collagen hydroxyprolination for GBM *versus* controls.
• Differential regulation of O-glycopeptides for proteoglycans for GBM *versus* controls.
• Increase in glycotransferase and glycosidase enzymes for GBM *versus* controls.

## Graphical Abstract

**Figure:**
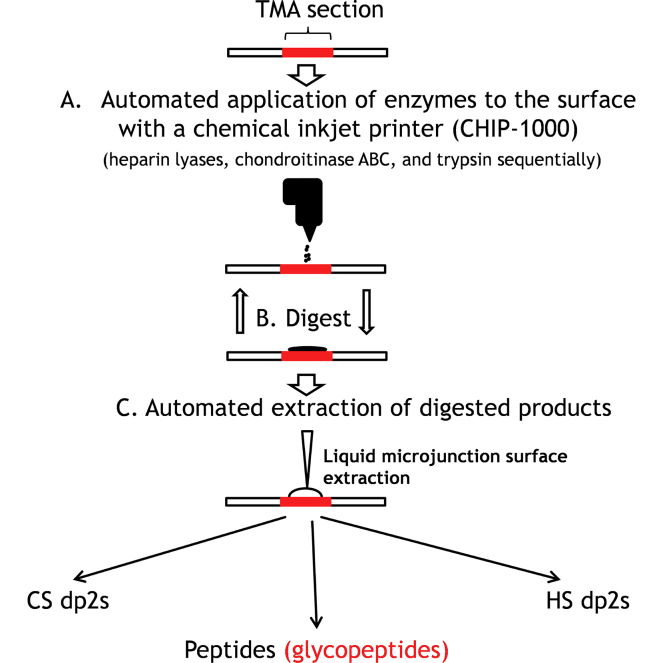

## In Brief

Glioblastoma (GBM) is the most malignant human brain tumor. It is critical to understand the molecular mechanisms in GBM and identify clinical markers. Extracellular matrix or matrisome is implicated in important neurological processes in brain cancer. But less attention has been paid to matrisomal changes and related glycosylation in GBM. In this manuscript, we performed an in-depth matrisomal, glycomic, and glycoproteomic analysis of control and GBM samples to identify key alterations in matrisomal components, strengthening our knowledge of GBM pathology.

Glioblastoma (GBM), the most common and malignant primary brain tumor in human adults (1), has a median survival time of fewer than 15 months with traditional treatment, including surgical resection, radiotherapy, and chemotherapy (2). In the United States, roughly 12,000 new cases are reported every year (3). GBM is characterized by the aberrant activation of multiple receptor tyrosine kinase (RTK) signaling pathways and poor prognosis. Therapies targeting RTK signaling pathways have achieved only limited clinical success (4). WHO grade IV diffuse glioma have traditionally been referred to as GBM, yet they are comprised of two molecularly and clinically distinct diseases-based presence or absence of mutations in isocitrate dehydrogenase 1 (IDH1) or isocitrate dehydrogenase 2 (IDH2). While IDH-wildtype (IDH-WT) tumors comprise the vast majority of cases, the IDH-mutant tumors are an important subset, and the vast majority of lower-grade diffuse glioma harbor IDH-mutations (5). The mutations in IDH1 or IDH2 result in increased production and accumulation of the oncometabolite 2-hydroxyglutarate with resulting profound metabolic effects on the cell (6, 7). GBM genomics expression studies identified three distinct transcriptional subclasses (classical, CLA; proneural, PRO; and mesenchymal, MES) characterized by abnormalities in epidermal growth factor receptor (EGFR), platelet-derived growth factor receptor alpha (PDGFRA), IDH1, and neurofibromin 1 (8, 9). The CLA subtype is associated with EGFR amplification, phosphatase and tensin homolog deleted on Chromosome 10 loss, and cyclin dependent kinase inhibitor 2A loss. The PRO subtype is characterized by alterations of PDGFRA and also includes the less common IDH-mt GBM. The MES subtype displays a high frequency of neurofibromin 1 mutation. In conclusion, GBM is a highly heterogeneous tumor type.

Proteoglycans (PGs) consist of a core protein and covalently attached linear polysaccharides known as glycosaminoglycans (GAGs). There is growing interest in documenting the roles of GAGs, PGs, and their binding partners that underpin biosynthetic and degrading enzymes in brain cancer and neurodegeneration (10, 11). Heparan sulfate proteoglycans (HSPGs), chondroitin sulfate proteoglycans (CSPGs), and hyaluronan/hyaluronic acid are the most abundant constituents of brain extracellular matrix (ECM) (referred to here as the matrisome) (12), which has been implicated in processes including proliferation, differentiation, adhesion, and cell survival in brain cancer and other neurological conditions (13, 14). Specifically, HSPGs and CSPGs are reported to be involved in the regulation of many RTK signaling pathways and to participate in cell–matrisome interaction related to brain cancers (15, 16, 17, 18, 19, 20, 21). HSPG and the extracellular HS modifying enzyme sulfatase 2 (Sulf2) have been implicated in the tumorigenesis of GBM (15). A comprehensive genomic analysis was conducted with data from The Cancer Genome Atlas (TCGA) (10) to better understand the alterations of PGs and their modifying enzymes for GBM. The study identified PG core proteins and GAG-modifying enzymes that were differentially expressed in GBM relative to normal brain tissues. For example, the mRNA expression level for the majority of glypicans, syndecans, and matrisome molecules, including brevican, neurocan, and versican, was significantly increased in GBM. Interestingly, GBM subtype-specific expression patterns were also observed from GAG-modifying enzymes, including Sulf1 and Sulf2, which were reduced for neural and CLA subtypes and increased for PRO and MES subtypes. A recent proteogenomic and metabolomics characterization of human GBM identified matrisome organization and collagen formation among the enriched pathways, highlighting the importance of the further study of the roles of matrisome organization in GBM (22).

We sought to acquire a comprehensive understanding of the biological functions of ECM, also called the matrisome, and its components, including PGs and GAGs in GBM tumorigenesis, and to identify potential biomarker candidates. To this end, we studied the alterations of GAGs (HS and CS), the core proteins of PGs, and other matrisomal proteins in GBM subtypes *versus* control human brain tissue samples. Additionally, we scrutinized the proteomics data to acquire in-depth site-specific glycoproteomic profiles of the GBM subtypes that will assist in identifying specific glycosylation changes in GBM. Taken together, this study aims to achieve a better understanding of the underlying molecular mechanisms in GBM and clinical markers for early detection or prognostic biomarkers of GBM that may have therapeutic potential as drug targets

## Experimental Procedures

### Materials

Chondroitinase ABC enzyme was from Sigma-Aldrich. Heparan sulfate disaccharides and chondroitin sulfate disaccharides were purchased from Celsus Laboratories. Heparin lyases I, II, and III were generous gifts from Prof. Jian Liu (UNC Eshelman School of Pharmacy). GlycanPac AXH-1 columns (2.1 mm∗15 cm and 300 μm∗15 cm) were generous gifts from ThermoFisher Scientific. Trypsin gold, mass spectrometry grade was from Promega Corp.

### Experimental Design and Statistical Rationale

In total, 43 tissue microarray (TMA) cores were used; 39 consisted of GBM subtypes (11 CLA, 9 MES, and 19 PRO) and four control cores. The control cores were from a single patient and corresponded to nonneoplastic regions (white matter and cortex) of the resected tissue and were not independent of one another. There was no neural subtype included in this work. For each molecular class, one biological replicate as a single LC-MS/MS run was performed. Although a limited number of controls were available, all results were subjected to multiple test corrections. All the samples were handled for sample processing and acquired on the instrument (for both glycomics and proteomics) at the same time to avoid any sample handling or instrument bias. Standard disaccharides, peptide retention time mixtures, and blanks were run routinely in between the samples. In addition, the disaccharide and peptide samples were spiked with internal controls. The subtypes were blinded, and all samples were acquired on the instrument randomly. Further, the peaks QC function of the PEAKS Studio X+ software package (Bioinformatics Solutions, Inc) was used as a quality control (QC) check for the data. supplemental Fig. S1*A* shows peaks QC plots that show protein, peptide, MS1, and MS/MS trends among 43 samples were very similar, indicating the high quality of the data acquired. In addition, supplemental Fig. S1, *B*–*D* shows representative extracted ion chromatograms (EICs) of spiked peptide retention time calibration mix for proteomic analysis and disaccharide standards for HS and CS glycomic analysis, respectively. All spiked internal standards were reproducible with respect to their retention times and intensities.

### Tumor Sample Information

GBM was identified from records in the UCSF Brain Tumor Center Biorepository and the Division of Neuropathology, Department of Pathology, at UCSF. While referred to as WHO grade IV GBM, IDH-WT, and GBM, IDH-mutant, as per the WHO 2016 classification (23), it is recognized that going forward these tumors will be known as GBM, IDH-WT, CNS WHO grade 4, and astrocytoma, IDH-mutant, CNS WHO grade 4, respectively, per the CNS WHO 2021 (24). The ethics approval number for the use of de-identified human biospecimens is 10-01318. These studies were in accordance with the ethical standards of the institutional research committee and with the 1964 Helsinki declaration and its later amendments. The transcriptional subgroup was determined based upon NanoString analysis of total RNA as previously described (25), and tumors were assigned GBM subtypes based on genome patterns (8): 11 CLA, nine MES, and 19 PRO. Briefly, For NanoString analysis, total RNA was isolated from tumor cores (2- to 3 1-mm cores) from formalin-fixed paraffin-embedded blocks containing >75% tumor cells as determined by H&E staining, according to the manufacturer’s protocol (RNEasy FFPE kit; Qiagen). Concentrations were determined using NanoDrop ND-1000 spectrophotometer (NanoDrop Technologies), and RNA integrity was assessed using an Agilent 2100 Bioanalyzer (Agilent Technologies). A custom code set was generated, and probes for the analysis were synthesized by NanoString technologies. The dataset included probes for 14 genes of interest, including five PRO genes [delta like canonical notch ligand 3 (DLL3), neural cell adhesion molecule 1 (NCAM1), oligodendrocyte transcription factor 2 (OLIG2), SRY-box transcription factor 9 (SOX9), and SRY-box transcription factor 2 (SOX2)], three MES genes [chitinase 3 like 1 (CHI3L1), TIMP metallopeptidase inhibitor 1 (TIMP1), and CD44], and six normalizing genes [actin beta (ACTB), beta-2-microglobulin (B2M), glyceraldehyde-3-phosphate dehydrogenase (GAPDH), RNA polymerase II subunit A (POLR2A), succinate dehydrogenase complex flavoprotein subunit A (SDHA), and TATA-box binding protein (TBP)]. RNA (200 ng) was analyzed with the NanoString nCounter Analysis System at NanoString Technologies according to the manufacturer’s protocol (NanoString Technologies). GBM exhibits both intertumoral and intratumoral heterogeneity. In selecting regions of interest for analysis, for each case, all molecular information and formalin-fixed paraffin-embedded cases as clinical H&E-stained slides from the resection were reviewed by a board-certified neuropathologist (J.J.P.) and two representative regions of high tumor (dense tumor regions) content or “central” tumor were selected for each case to be included in the University of California, San Francisco Brain Tumor Center Biorepository. These regions were used to generate TMAs for profiling and used for the isolation of RNA for transcriptional analysis. The sampling of multiple tumor regions from regions with high-tumor content tends to sample tumor clones that can help drive tumor progression (26). The analysis of different regions of the same tumor is an area of great interest; however, it was not the focus of this manuscript and would be interesting to explore in the future. An example of representative images of H&E-stained sections of TMA cores from 08257(MES) and 08306 (PRO) (supplemental Fig. S2, *A* and *B*) highlight tumor cell density in regions of central tumor. TMA cores region included in each core include tumor cells, vasculature, and eosinophilic regions containing both cell processes and ECM. Table 1 provides detailed information on the cases, including disease type, GBM subtype, IDH1 and EGFR status, patient age, sex (M; male, F; female), treatment, anatomic location, and tumor region. Supplemental Table S1 provides information, including raw file names and peak database search numbers for proteomics analysis.

**Table 1:** Detailed information on the cases, including disease type, GBM subtype, IDH1 and EGFR status, patient age, sex (M; male, F; female), treatment, anatomic location, and tumor region

UCSF code	2016 WHO integrated diagnosis	EGFR	Subtype	Sex	Age	Newly diagnosed (N) or recurrent (R)	Treatment	Anatomic location	Tumor region
06969	Glioblastoma, IDH-wildtype	Unknown	Mesenchymal	M	59	N	None	Parietal	Central
07034	Glioblastoma, IDH-wildtype	Amplified	Classical	F	51	N	None	Parietal	Central
07686	Glioblastoma, IDH-wildtype	Amplified	Mesenchymal	F	46	N	None	Temporal	Central
07880	Glioblastoma, IDH-wildtype	Amplified	Classical	M	40	N	None	Frontal	Central
08216	Glioblastoma, IDH-wildtype	Unknown	Classical	M	73	N	None	Frontal	Central
08257	Glioblastoma, IDH-wildtype	Unknown	Mesenchymal	F	55	N	None	Parietooccipital	Central
08306	Glioblastoma, IDH-wildtype	Unknown	Proneural	M	79	N	None	Frontal	Central
08318	Glioblastoma, IDH-wildtype	Unknown	Proneural	M	60	N	None	Temporal	Central
08345	Glioblastoma, IDH-wildtype	Unknown	Proneural	M	66	N	None	Frontal	Central
08351	Glioblastoma, IDH-wildtype	Unknown	Proneural	F	67	N	None	Occipital	Central
8386	Glioblastoma, IDH-wildtype	Unamplified	Proneural	M	48	N	None	Frontal	Central
08624	Glioblastoma, IDH-wildtype	Unamplified	Proneural	M	63	N	None	Parietal	Central
08858	Glioblastoma, IDH-wildtype	Unamplified	Mesenchymal	F	61	N	None	Temporal	Central
09259	Glioblastoma, IDH-wildtype	Unamplified	Proneural	M	73	N	None	Frontotemporal	Central
09295	Glioblastoma, IDH-wildtype	Amplified	Classical	F	60	N	None	Temporoparietal	Central
09459	Glioblastoma, IDH-wildtype	Amplified	Classical	F	70	N	None	Parietal	Central
08061	Glioblastoma, IDH-wildtype	Amplified	Classical	M	44	R	None	Frontal	Central
5705	Glioblastoma, IDH-wildtype	Amplified	Mesenchymal	F	51	R	XRT+TMZ	Frontal	Central
06884	Glioblastoma, IDH-mutant	Unamplified	Classical	F	31	N	None	Frontal	Central
07491	Glioblastoma, IDH-mutant	Unamplified	Proneural	F	36	N	None	Occipital	Central
07884	Glioblastoma, IDH-mutant	Unamplified	Proneural	M	33	N	None	Frontal	Central
08354	Glioblastoma, IDH-mutant	Unknown	Proneural	F	20	N	None	Parietal	Central
7499	Glioblastoma, IDH-wildtype	Unamplified	Mesenchymal	F	71	N	None	Temporal	Central
9418	Glioblastoma, IDH-wildtype	Unknown	Classical	F	47	N	None	Temporoparietal	Central

Controls						Description			Clinical indication
Control 1	Mild reactive astrogliosis	Unknown		F	37	White matter	NA	Frontotemporal	Medically refractor seizures
Control 2	Mild reactive astrogliosis	Unknown		F	37	Cerebral cortex	NA	Frontotemporal	Medically refractor seizures

Abbreviations: GBM, glioblastoma; IDH1, isocitrate dehydrogenase 1.

### Automated Enzyme Addition to Tissue Surfaces With Inkjet Chip Printer

A chemical inkjet chip printer (Shimadzu CHIP-1000) was used for printing enzymes to tissue surfaces (the workflow is shown in supplemental Fig. S3). This device is capable of applying minimal 500 pL solutions to tissue surfaces. The printer scans an image of the TMA slide, which enables the user to designate the cores to be analyzed. Next, 1 μl enzyme solution, sufficient to cover the entire core surface, was applied to each designated core using a user-defined printing pattern. To ensure exhaustive digestion, the enzyme printing process was repeated four times. The time interval for the repeated enzyme printing was 2 h. After the fourth enzyme printing, the TMA slides were incubated in a humidified digestion chamber at 37 °C, overnight. The detailed *on-slide* digestion protocol could be obtained from our previous publications (27, 28). Briefly, a mixture of 0.5 mU/μl heparin lyases I, II, and III was applied first, followed by overnight incubation. Second, a 1 mU/μl chondroitinase ABC enzyme solution was applied to the same spot. Finally, 100 ng/μl trypsin solution was applied to the spots.

### Automated Extraction of Digested Molecules From Tissue surfaces With Nanomate Robot

Liquid extraction surface analysis entails automated extraction of compounds from tissue surfaces with minimized disturbance to tissue. A liquid extraction surface analysis module has been developed for the widely used Advion Triversa Nanomate (Advion Inc) LC/MS sample manipulation robot. The robot took 1 μl extraction buffer and delivered the solution to the designated core to form a 1.5 μm liquid microjunction. The digested products were resolved in the liquid microjunction and extracted from the tissue surface. This extraction process was repeated 5 times to enable maximum sample recovery from tissue surfaces. The optimum extraction buffer for digested GAGs and peptides was 0.3% ammonium hydroxide and 10% ACN/90% water/0.1% formic acid solution, respectively.

### GAGs and Peptides Desalting

The GAG extractions were dried by vacuum centrifugation and desalted using an SEC column (Superdex peptide PC 3.2/30, GE Healthcare), using 25 mM ammonium acetate in 5% ACN (pH = 4.4) as the mobile phase at an isocratic flow 0.04 ml/min for 60 min. The disaccharides eluted between 35 and 45 min and were detected using UV absorbance at 232 nm. The cleaned disaccharide samples were dried by vacuum centrifugation and stored at −20 °C until analyzed using LC-MS/MS. The extracted peptides were dried in vacuum centrifugation and passed through C-18 zip tips (Thermo Fisher Scientific); the cleaned peptides were eluted using 60% ACN/water/0.1% TFA and dried by vacuum centrifugation. The cleaned peptides were further stored at −20 °C until analyzed using LC-MS/MS.

### Liquid Chromatography-Tandem Mass Spectrometry (LC-MS/MS) Glycomics Analysis

HS and CS disaccharides were analyzed using negative ionization mode electrospray LC-MS/MS as previously described (27, 29, 30). HILIC resins were de-packed from three 2.1 mm∗15 cm chromatography columns (Thermo Dionex Glycan Pac AXH-1, Thermo Dionex Accucore Amide 150, and Waters BEH HILIC) and tested for HS disaccharides separation. Nano-HILIC columns (100 μm∗ 15 cm) were packed in-house. The columns were mounted on Waters NanoAcquity chromatograph coupled to a Q-Exactive plus mass spectrometer (ThermoFisher Scientific). Tandem MS using higher energy collision-induced dissociation with a normalized collision energy of 30 eV was conducted to discriminate D2A0 from D0A6 and D2S0 from D0S6. Mass spectra were acquired in the Orbitrap mass analyzer with one microscan per spectrum for both MS and MS/MS in negative ionization mode. The resolving power for MS and MS/MS was set at 70,000 and 17,500. The precursor ion isolation window was set to 2 u. The mobile phase A is 50 mM ammonium formate, pH 4.4, and the mobile phase B is 95% ACN/5% HPLC water. The flow rate was set at 0.6 ul/min, and a gradient was set at 92.5% B to 50% B in 30 min, followed by 50% B to 20% B in 5 min, and then equilibration to 92% B in 20 min. For CS disaccharide analysis, 300 μm∗15 cm column (packed with 1.9 μm Thermo Dionex Glycan Pac AXH- one resin, a generous gift from Thermo Dionex) was mounted to Waters Acquity chromatograph. It was interfaced to an LTQ-Orbitrap XL mass spectrometer (ThermoFisher Scientific). Tandem MS using CID with a normalized collision energy of 30 eV was conducted to discriminate D0a4 from D0a6. Mass spectra were acquired in the Orbitrap mass analyzer with one microscan per spectrum for both MS and MS/MS at negative mode. Resolving power for MS and MS/MS was set at 60,000 and 15,000, respectively. The isolation window for the targeted precursor is 3 u. The mobile phase A is 50 mM ammonium formate, pH 4.4, and the mobile phase B is 95% ACN/5% HPLC water. The LC was running at an isobaric gradient at 85%B with 7 μl/min. Each LC run was reduced to 20 min to maximize throughput. The relative and absolute abundance were determined using standard curves as previously described (30, 31).

### Liquid Chromatography-Tandem Mass Spectrometry Proteomics Analysis

Nano-LC-MS/MS separation was performed using a nanoAcquity UPLC (Waters Technology) and Q-Exactive Plus mass spectrometer (ThermoFisher Scientific). Reversed phased C-18 analytical (BEH C18, 150 μm × 100 mm) and trapping (180 μm × 20 mm) columns from Waters technology were used with a 120 min method with a gradient from 2 to 98% acetonitrile in 97 min, using 99% water/1% acetonitrile/0.1% formic acid as mobile phase A and 99% acetonitrile/1% water/0.1% formic acid as mobile phase B at a flow rate of 0.5 μl/min as previously described in (29). Data-dependent acquisition tandem MS was acquired in the positive ionization mode for the top 20 most abundant precursor ions. Full MS scans were acquired from *m/z* 350 to 1500 with 70,000 resolution using an automatic gain control target of 3e6 and maximum injection time (IT) of 50 ms. Dynamic exclusion (10 s) was enabled. The minimum threshold for precursor selection was set to 5 × 10^4^. Precursor ions were fragmented using a resolution of 17,500 with a maximum injection time of 45 ms and an automatic gain control value of 2e5 using higher energy collision-induced dissociation with a normalized collision energy of 27 V.

### Glycomics Data analysis Using Excel Spreadsheet

Student’s *t* tests were performed with two-tailed distribution using Microsoft Excel to test for alterations in the obtained HS and CS disaccharide profiles. Different concentrations (500 fmol, 1 pmol, 2 pmol, 5 pmol, and 10 pmol) of eight HS standard disaccharides (D0A0, D2A0, D0A6, D2A6, D0S0, D2SO, D0S6, and D2S6) (Iduron) and four CS disaccharides (D0a0, D0a4, D0a6, D0a10) were run on LC-MS as triplicates, for plotting a MS standard curve. The MS/MS standard curve was plotted for different ratios of HS isoforms (D2A0/D0A6 and D2SO/D0S6) and CS isoforms (D0a4/D0a6) as previously described (24). The area under the curve for EIC for HS and CS disaccharides from each sample were obtained from the raw LC-MS/MS data using qualitative analysis software (version B.06; Agilent Technologies). The obtained abundances for each disaccharide were first normalized to a spiked internal control (as indicated above) and then further normalized to a standard curve to obtain an absolute abundance of HS or CS disaccharides (fmol). A relative abundance was then calculated for each HS (supplemental File S1) and CS (supplemental File S2) disaccharides. For differentiating the isoforms, EIC (MS/MS) for diagnostic ions as indicated in (28) for each CS and HS disaccharide isoform was extracted, and abundance was obtained from manual area calculation. The obtained abundance was then used to calculate the percent relative abundance of each isoform which was further normalized using the MS/MS standard curve to obtain the percent absolute abundance of each HS or CS isoform.

### Protein Identification and Label-Free Quantification Using PEAKS Software

The raw LC-MS/MS data were converted into mzXML format using ProteoWizard msConvert (Version: 3.0.20210-c0fceb89a) (32). The data were searched using PeaksDB and PeaksPTM using Peaks Studio, version X+ (Bioinformatics Solutions, Inc) against the Uniprot/Swissprot database (release 2020_04) for homosapiens with a 1% false discovery rate and at least two unique peptides. False discovery rate (FDR) is estimated with the decoy fusion method, *i.e.*, an enhanced target-decoy method using Peaks X+ software. A 10-ppm error tolerance for the precursor (MS1) and 0.02 Da mass error tolerance for fragment ions (MS2) were specified. A maximum of three missed cleavages per peptide was allowed for the database search, permitting nontryptic cleavage at one end. Trypsin was specified as the enzyme and carbamidomethylation as a fixed modification. The samples were tested for QC using PEAKS QC analysis; no significant difference with respect to protein, peptide MS1, MS2 trends, retention time, and precursor mass tolerance was observed for the samples indicating the high quality of the presented data (supplemental Fig. S1). A peaksPTM search was queued after the peaksDB search, using advanced settings of a larger set of variable modifications, including hydroxylation P, oxidation M, hydroxylation K, hydroxylation-Hex K, hydroxylation-Hex-Hex K, HexNAc ST, HexHexNAc ST, phosphorylation STY, ubiquitination K, deamidation N, methoxy K, and nitrotyrosine Y. The final protein list generated was a combination of peaskDB and peaksPTM searches. The label-free quantification was achieved using the PEAKS Studio Quantification (PeaksQ)—label-free module with a setting of mass error tolerance of 10 ppm and a retention time shift tolerance of 2.0 min. The identified protein, protein–peptide, and peptide list from peaks PTM analysis are provided in supplemental File S3.

### Matrisome Analysis

The label-free exported “proteins” (supplemental File S4) and “protein–peptide” (supplemental File S5) comma-separated lists were filtered to include only matrisome components as identified by Naba *et al.* (33), supplemental File S6; matrisome proteins, and supplemental File S7; matrisome protein–peptide.

### Statistical Analysis and Visualization in RStudio and Microsoft Excel

The matrisome label-free quantified protein and their corresponding “protein–peptide” exports were used to compare the abundances of proteins and peptides between GBM and control samples and between GBM subtypes. Statistical analysis and visualization were carried out in RStudio (34). Heat maps of the log-transformed abundances of core matrisome components were generated using ggplot2. For statistical analysis, the compare means function was used with a FDR to correct for multiple comparisons (35). For visualization, boxplots of the log-transformed abundances of significantly differentially expressed proteins and peptides were generated. Further, the fold change and corresponding t-values were obtained by comparing normalized log-transformed protein and peptide abundances (FDR < 0.05) between various group comparisons, including GBM *versus* control, PRO/MES/CLA *versus* control, PRO *versus* MES, PRO *versus* CLA, and MES *versus* CLA analyses. The glycomics analysis was performed in Microsoft excel, and *p*-values were calculated by using heteroscedastic *t* test, and *p*-values < 0.05 were considered significant.

### GlycReSoft Analysis

Data were searched using the publicly available in-house GlycReSoft graphical user interface (desktop version) (36, 37) (https://github.com/BostonUniversityCBMS). A fasta glycopeptide hypothesis comprising the glycosylated and matrisomal proteins generated using UniProtKB (supplemental File S8) was used. A combinatorial glycan hypothesis was used to generate possible glycan compositions with the following rules: HexNAc 2-8, Hex 3-10, Fuc 0-5, NeuAc 0-4, HexNAc > Fuc, HexNAc - 1 > NeuAc. A list of identified total glycopeptides divided by sample number and intensity signal is provided in supplemental File S9. The identified glycopeptides corresponded to *N*-glycosites and Ser/Thr-*O*-glycosites. Three glycopeptides that corresponded to paucimannosidic N-glycosylation were detected in a few of the samples. These did not pass the RAMZIS score threshold as described in the next paragraph.

### Glycopeptide Similarity Analysis

The glycopeptides identified by GlycReSoft (supplemental File S9) were analyzed using RAMZIS, an R toolkit for assessing glycoproteomic data using contextual similarity (38). RAMZIS generates bootstrapped datasets to simulate comparisons that provide information on the consistency of the sample groups, their ability to be used in the comparison, the likelihood of a systemic difference, and the best candidates for further analysis. Using RAMZIS, each sample group was assessed for internal consistency and put into pairwise comparisons: the control was compared to an aggregate of the CLA, MES, and PRO subtypes as well as to each subtype on its own; the subtypes were similarly compared against each other. The following standards were used within the RAMZIS framework. For a dataset to be used in a comparison, it had to have an internal confidence score greater than 2, indicating that the internal similarity simulations, or internal similarity distribution, were on average more than two weighted standard deviations from the mean of the test similarity distribution. The test similarity distribution was only considered to reliably emulate the comparison if the original data comparison, or the Observed Similarity, was within the central two quartiles of the test distribution as a percentile or within two standard deviations of the test distribution mean; if it fell outside this region, then RAMZIS was not considered to emulate the comparison well. The general comparison was considered likely to find a systemic difference between the glycopeptide distributions if the test similarity distribution and null similarity distribution, the simulation of the null hypothesis of equivalent glycopeptide distributions, had a false positive overlap less than 0.05 or 5% and a false negative overlap less than 0.20 or 20%. Individual glycopeptides were reported with QCs that indicated if there were more than 25% overlap between their test and internal distributions, indicating the combined false positive and false negative rates; they were ranked according to the test distributions’ separation to the respective null distributions with lower scores indicating a greater likelihood of a difference existing. These rankings were then assessed manually.

### Gene Set Enrichment and Network Topology Analysis

The matrisome (supplemental File S10) differentially expressed protein and peptide lists (FDR < 0.05) for GBM *versus* control, and all subtypes comparisons were analyzed using WEB-based GEne SeT AnaLysis Toolkit (WebGestalt; http://www.webgestalt.org/) (39), for gene set enrichment (GSEA) and network topology analysis (NTA) to identify enriched GO terms (biological process, cellular component, molecular function, pathways, and networks specific to control and GBM subtypes).

## Results

### GAG Expression

**Fig. 1:**
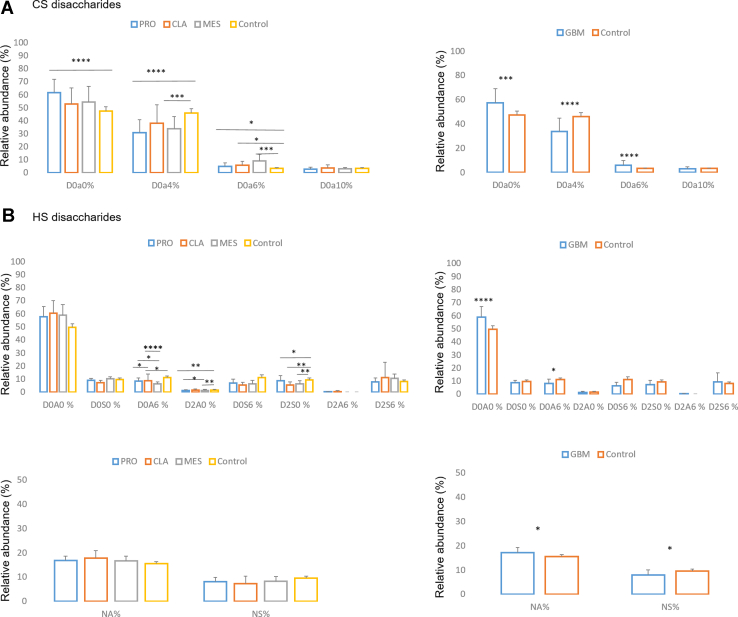
**Disaccharides relative abundance.** The relative abundance (%) for CS (*A*) and HS (*B*) disaccharides for proneural (PRO), mesenchymal (MES), and classical (CLA) GBM subtype, and control samples (*left*) and for GBM total (average of all subtypes) and control samples (*right*). For HS disaccharides %, relative abundance of *N*-acetylated (NA) *versus N*-sulfated (NS) is also provided for GBM subtypes and control (*bottom left*), and GBM total and control (*bottom right*) samples. Nomenclature for the Lawrence code are provided in supplemental Table S1 (∗*p* ≥ 0.05, ∗∗*p* ≥ 0.001, ∗∗∗*p* ≥ 0.0001, ∗∗∗∗*p* ≥ 0.00001) (mean ± SD). CS, chondroitin sulfate; GBM, glioblastoma; HS, heparan sulfate.

Supplemental Table S2 shows the Lawrence code nomenclature used here for the CS and HS disaccharides. The relative abundances calculation for CS (supplemental File S1) and HS disaccharides (supplemental File S2) are shown in Figure 1, *A* and *B*, respectively. The most abundant CS disaccharide for GBM and control samples was unsulfated D0a0, which was significantly elevated for GBM samples, specifically PRO subtype, as compared to controls. For the monosulfated disaccharides, 4-*O*-sulfated D0a4 was significantly decreased in GBM samples, specifically PRO and MES subtypes relative to the controls. On the other hand, 6-*O*-sulfated D0a6 was increased for GBM compared to control samples, with a notable increase in 6-*O* sulfation of CS for MES observed. For HS disaccharides, *N*-acetylated disaccharides were more abundant than *N*-sulfated for both GBM and control samples, and *N*-acetylated disaccharides were significantly increased, and *N*-sulfated disaccharides were significantly decreased for GBM *versus* control samples. Moreover, D0A0 disaccharide was dramatically the most abundant HS disaccharide in both GBM and control samples and significantly increased for GBM compared to control samples. Conversely, D0A6, *i.e.*, HS 6-*O* sulfation, was significantly decreased for GBM compared to controls, which was an opposite expression to CS 6-*O* GalNAc sulfation. Some other significant differences for subtypes *versus* control HS disaccharides were observed, namely for D0A6, D2A0, and D2S0 disaccharides.

### Proteomics Analysis and Matrisome List Extraction

From the label-free proteomics analysis, 1280 proteins with ≥2 unique peptides (supplemental File S4) were quantified that corresponded to 28,522 peptides (spplemental File S5). The quantified protein list was then compared to the brain matrisome protein list from Naba *et al.* (33), and a total of 146 matrisome-related proteins were filtered (supplemental File S6), comprising 75 core matrisome constituents, including PGs, glycoproteins, and collagens, and 71 matrisome-associated proteins including regulators, affiliated proteins, and secreted factors. Table 2 shows accession and matrisome type for the 146 matrisomal proteins identified in this study.

**Table 2:** List of core matrisome (left) and matrisome-associated proteins (right) as observed in this study

Accession	Matrisome type
P01009|A1AT_HUMAN	ECM regulators
P02750|A2Gl_HUMAN	ECM glycoproteins
P08697|A2AP_HUMAN	ECM regulators
P01023|A2MG_HUMAN	ECM regulators
P01011|AACT_HUMAN	ECM regulators
O14672|ADA10_HUMAN	ECM regulators
Q9P0K1|ADA22_HUMAN	ECM regulators
Q8IUX7|AEBP1_HUMAN	ECM glycoproteins
O00468|AGRIN_HUMAN	ECM glycoproteins
P01019|ANGT_HUMAN	ECM regulators
P01008|ANT3_HUMAN	ECM regulators
P04083|ANXA1_HUMAN	ECM-affiliated proteins
P07355|ANXA2_HUMAN	ECM-affiliated proteins
P09525|ANXA4_HUMAN	ECM-affiliated proteins
P08758|ANXA5_HUMAN	ECM-affiliated proteins
P08133|ANXA6_HUMAN	ECM-affiliated proteins
P20073|ANXA7_HUMAN	ECM-affiliated proteins
P50995|ANX11_HUMAN	ECM-affiliated proteins
P02760|AMBP_HUMAN	ECM regulators
Q15582|BGH3_HUMAN	ECM glycoproteins
P07858|CATB_HUMAN	ECM regulators
P07339|CATD_HUMAN	ECM regulators
P08311|CATG_HUMAN	ECM regulators
P09668|CATH_HUMAN	ECM regulators
Q9UBR2|CATZ_HUMAN	ECM regulators
Q6YHK3|CD109_HUMAN	ECM regulators
Q5KU26|COL12_HUMAN	ECM-affiliated proteins
P02452|CO1A1_HUMAN	Collagens
P08123|CO1A2_HUMAN	Collagens
P02458|CO2A1_HUMAN	Collagens
P02461|CO3A1_HUMAN	Collagens
P02462|CO4A1_HUMAN	Collagens
P08572|CO4A2_HUMAN	Collagens
P20908|CO5A1_HUMAN	Collagens
P05997|CO5A2_HUMAN	Collagens
P25940|CO5A3_HUMAN	Collagens
P12109|CO6A1_HUMAN	Collagens
P12110|CO6A2_HUMAN	Collagens
P12111|CO6A3_HUMAN	Collagens
P12107|COBA1_HUMAN	Collagens
Q99715|COCA1_HUMAN	Collagens
Q05707|COEA1_HUMAN	Collagens
Q07092|COGA1_HUMAN	Collagens
P39060|COIA1_HUMAN	Collagens
Q2UY09|COSA1_HUMAN	Collagens
P13611|CSPG2_HUMAN	Proteoglycans
Q6UVK1|CSPG4_HUMAN	ECM-affiliated proteins
P02746|C1QB_HUMAN	ECM-affiliated proteins
P01034|CYTC_HUMAN	ECM regulators
P04080|CYTB_HUMAN	ECM regulators
P15502|ELN_HUMAN	ECM glycoproteins
Q9Y6C2|EMIL1_HUMAN	ECM glycoproteins
Q9BXX0|EMIL2_HUMAN	ECM glycoproteins
P23142|FBLN1_HUMAN	ECM glycoproteins
Q9UBX5|FBLN5_HUMAN	ECM glycoproteins
P00488|F13 A_HUMAN	ECM regulators
P35555|FBN1_HUMAN	ECM glycoproteins
P05230|FGF1_HUMAN	ECM glycoproteins
P02671|FIBA_HUMAN	ECM glycoproteins
P02675|FIBB_HUMAN	ECM glycoproteins
P02679|FIBG_HUMAN	ECM glycoproteins
P02751|FINC_HUMAN	ECM glycoproteins
P35052|GPC1_HUMAN	ECM-affiliated proteins
P51610|HCFC1_HUMAN	Secreted factors
P02790|HEMO_HUMAN	ECM-affiliated proteins
P05546|HEP2_HUMAN	ECM regulators
Q8NDA2|HMCN2_HUMAN	ECM glycoproteins
Q86YZ3|HORN_HUMAN	Secreted factors
P10915|HPLN1_HUMAN	Proteoglycans
Q9GZV7|HPLN2_HUMAN	Proteoglycans
P04196|HRG_HUMAN	ECM regulators
P18065|IBP2_HUMAN	ECM glycoproteins
P24593|IBP5_HUMAN	ECM glycoproteins
Q16270|IBP7_HUMAN	ECM glycoproteins
P05155|IC1_HUMAN	ECM regulators
P30740|ILEU_HUMAN	ECM regulators
P19827|ITIH1_HUMAN	ECM regulators
P19823|ITIH2_HUMAN	ECM regulators
Q14624|ITIH4_HUMAN	ECM regulators
Q86UX2|ITIH5_HUMAN	ECM regulators
P01042|KNG1_HUMAN	ECM regulators
P09382|LEG1_HUMAN	ECM-affiliated proteins
P17931|LEG3_HUMAN	ECM-affiliated proteins
O00182|LEG9_HUMAN	ECM-affiliated proteins
P49257|LMAN1_HUMAN	ECM-affiliated proteins
P24043|LAMA2_HUMAN	ECM glycoproteins
Q16363|LAMA4_HUMAN	ECM glycoproteins
O15230|LAMA5_HUMAN	ECM glycoproteins
P07942|LAMB1_HUMAN	ECM glycoproteins
P55268|LAMB2_HUMAN	ECM glycoproteins
P11047|LAMC1_HUMAN	ECM glycoproteins
Q9Y6N6|LAMC3_HUMAN	ECM glycoproteins
O95970|LGI1_HUMAN	ECM glycoproteins
Q8N145|LGI3_HUMAN	ECM glycoproteins
P51884|LUM_HUMAN	Proteoglycans
O00339|MATN2_HUMAN	ECM glycoproteins
P20774|MIME_HUMAN	Proteoglycans
P50281|MMP14_HUMAN	ECM regulators
O14594|NCAN_HUMAN	Proteoglycans
P14543|NID1_HUMAN	ECM glycoproteins
Q14112|NID2_HUMAN	ECM glycoproteins
P10451|OSTP_HUMAN	ECM glycoproteins
P13674|P4HA1_HUMAN	ECM regulators
P36955|PEDF_HUMAN	ECM regulators
P98160|PGBM_HUMAN	Proteoglycans
Q96GW7|PGCB_HUMAN	Proteoglycans
P21810|PGS1_HUMAN	Proteoglycans
P07585|PGS2_HUMAN	Proteoglycans
P00747|PLMN_HUMAN	ECM regulators
O60568|PLOD3_HUMAN	ECM regulators
Q9UIW2|PLXA1_HUMAN	ECM-affiliated proteins
O43157|PLXB1_HUMAN	ECM-affiliated proteins
O15031|PLXB2_HUMAN	ECM-affiliated proteins
Q15063|POSTN_HUMAN	ECM glycoproteins
P10619|PPGB_HUMAN	ECM regulators
P51888|PRELP_HUMAN	Proteoglycans
Q92626|PXDN_HUMAN	ECM glycoproteins
P60903|S10AA_HUMAN	Secreted factors
P31949|S10AB_HUMAN	Secreted factors
Q99584|S10AD_HUMAN	Secreted factors
Q96FQ6|S10AG_HUMAN	Secreted factors
P23297|S10A1_HUMAN	Secreted factors
P26447|S10A4_HUMAN	Secreted factors
P06703|S10A6_HUMAN	Secreted factors
P05109|S10A8_HUMAN	Secreted factors
P06702|S10A9_HUMAN	Secreted factors
P04271|S100 B_HUMAN	Secreted factors
O75056|SDC3_HUMAN	ECM-affiliated proteins
P50454|SERPH_HUMAN	ECM regulators
P35237|SPB6_HUMAN	ECM regulators
P50453|SPB9_HUMAN	ECM regulators
Q9HCB6|SPON1_HUMAN	ECM glycoproteins
P09486|SPRC_HUMAN	ECM glycoproteins
Q14515|SPRL1_HUMAN	ECM glycoproteins
P24821|TENA_HUMAN	ECM glycoproteins
Q92752|TENR_HUMAN	ECM glycoproteins
P22105|TENX_HUMAN	ECM glycoproteins
P21980|TGM2_HUMAN	ECM regulators
P00734|THRB_HUMAN	ECM regulators
Q9GZM7|TINAL_HUMAN	ECM glycoproteins
P35030|TRY3_HUMAN	ECM regulators
P04004|VTNC_HUMAN	ECM glycoproteins
Q6PCB0|VWA1_HUMAN	ECM glycoproteins
O00534|VMA5A_HUMAN	ECM glycoproteins
P04275|VWF_HUMAN	ECM glycoproteins

Abbreviation: ECM, extracellular matrix.

### Matrisome Changes

**Fig. 2:**
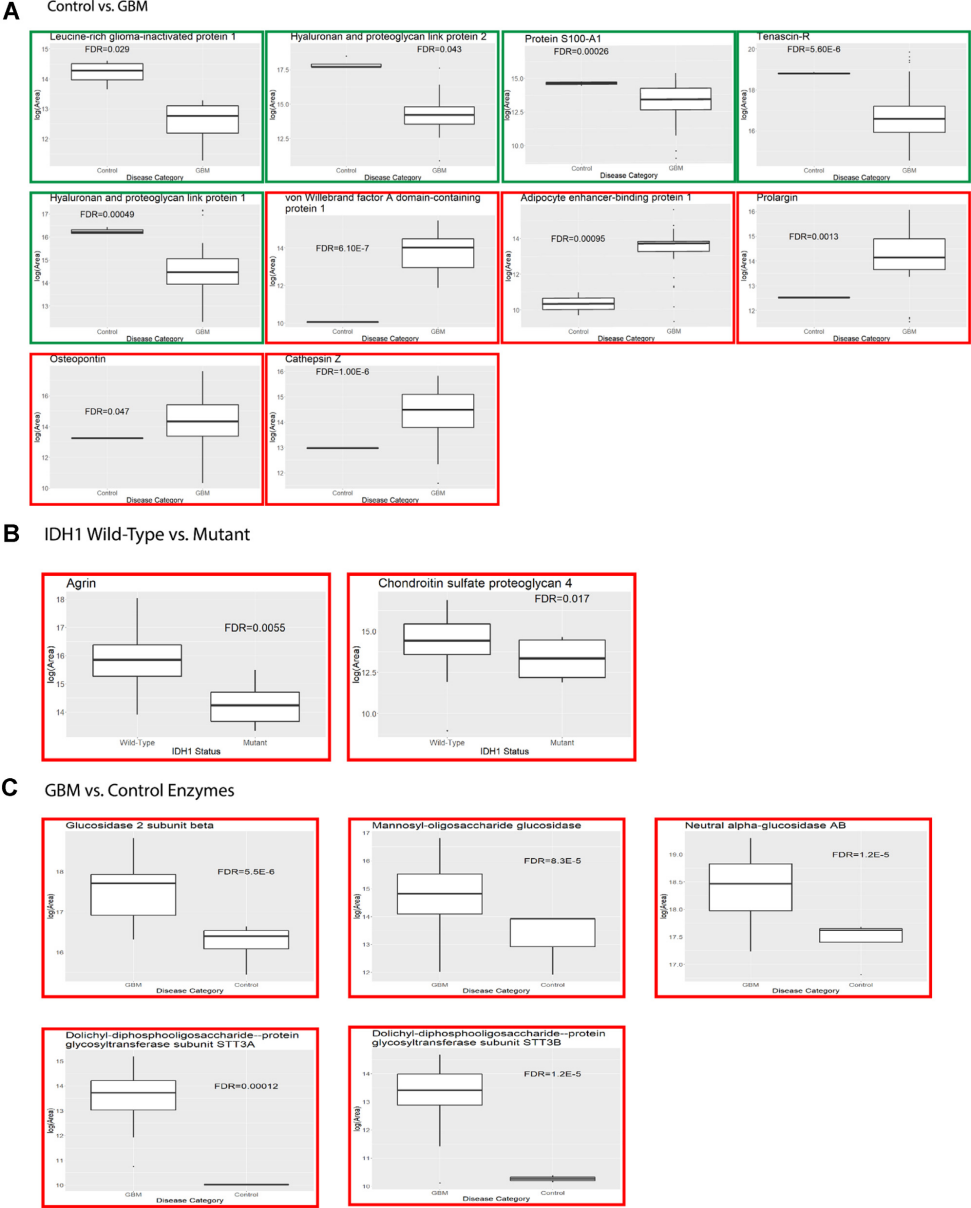
**Differential expressed proteins.** Box plots representing normalized abundances for *A*. Top five increased (*red box*) and decreased (*green box*) proteins (FDR < 0.05) from Table 3 for GBM *versus* control. *A*, GBM *versus* control. *B*, wildtype *versus* IDH1 mutant. *C*, altered glycosyltransferase and glycosidase enzyme for GBM *versus* control. The comparisons between normalized log-transformed abundances were corrected for multiple comparisons. FDR, false discovery rate; GBM, glioblastoma; IDH1, isocitrate dehydrogenase 1.

The quantified matrisomal protein and peptide list (supplemental Files S6 and S7) were analyzed in-depth to identify matrisomal changes within the three GBM subtypes (PRO *versus* CLA, PRO *versus* MES, CLA *versus* MES) and GBM and GBM subtypes versus control. A number of proteins and peptides (supplemental File S10) were observed to be differentially regulated (FDR < 0.05) between various group comparisons with a log2 fold change threshold of >1 or < −1 as previously used for brain cohort studies (40). The top ten significantly increased or decreased proteins for GBM *versus* control, and their fold change values for GBM subtype *versus* control comparisons are presented in Table 3, and the normalized log-transformed abundances for top five increased and decreased proteins for GBM *versus* control are presented as box plots in Figure 2*A* and for GBM subtypes *versus* control in supplemental Fig. S4. For GBM, 78 proteins were significantly increased, while eight proteins were decreased compared to the control samples. On the subtype level, PRO and MES subtype showed 41 and 25 proteins increased, and one and four proteins decreased, respectively, when compared to the control samples. Interestingly, the CLA subtype showed 12 proteins to be significantly increased while no proteins were significantly reduced as compared to the control samples. The fold changes for differentially expressed proteins in the GBM subtype *versus* control were observed to be higher than the GBM (all subtypes together) *versus* control comparisons. While the majority of the proteins were observed in higher abundances for GBM or GBM subtype samples relative to controls, only eight proteins were observed in higher abundances in controls, including leucine-rich glioma-inactivated protein 1 (LGI1), tenascin R, hyaluronan proteoglycan link protein 1 and 2 (HAPLN1, HAPLN2), laminin subunit beta-2 (LAMB2), and decorin (DCN).

**Table 3:** Differentially expressed proteins (FDR < 0.05) for top 10 increased (fold change > 1) and decreased proteins (fold change < 1) for GBM *versus* control and their fold change values for GBM subtype (PRO, MES, CLA) *versus* control comparisons

Proteins	Fold change			
	GBM *versus* Control	PRO *versus* Control	MES *versus* Control	CLA *versus* Control
Top-10 increased proteins				
Von Willebrand factor A domain-containing protein 1	1.6	190.1	−	245.5
Adipocyte enhancer-binding protein 1	1.4	38.5	−	−
Prolargin	1.3	27.6	−	−
Osteopontin	1.3	−	−	
Cathepsin Z	1.3	19.6	26.8	−
Collagen alpha-1(XVI) chain	1.2	14.6	11.1	−
Periostin	1.2	−	−	−
Kininogen-1	1.2	12.5	−	15.6
Fibulin-5	1.2	−	−	−
Collagen alpha-2(VI) chain	1.2	−	−	−
Top-10 Decreased Proteins				
Leucine-rich glioma-inactivated protein 1	−1.4	−		−
Hyaluronan and proteoglycan link protein 2	−1.2	−		−
Protein S100-A1	−1.1	−		−
Tenascin-R	−1	−3.2	−4.8	−
Hyaluronan and proteoglycan link protein 1	−1	−	−3.8	−
EMILIN-2	−1	−	−	−
Laminin subunit beta-2	−1	−	−2.4	−
Decorin	−1	−	−2.7	−

Abbreviations: CLA, classical; GBM, glioblastoma; MES, mesenchymal; PRO, proneural.

Among the proteins observed at higher abundances in GBM relative to controls, we observed von Willebrand factor A domain-containing protein 1 (VWA1) to be about two-hundred-fold higher for PRO and CLA subtypes (supplemental Fig. S4*A*). Notably, while tenascin R was increased for controls, tenascin (TNC) was increased for GBM samples (supplemental File S10). Several PGs, including prolargin (PRELP), agrin (AGRN), and biglycan (BGN), were elevated in GBM samples relative to controls. However, the abundances of lecticans such as versican (VCAN) and brevican (BCAN) were similar in GBM subtypes versus controls.

A number of blood coagulation related proteins, including VWA1, antithrombin III (ANT3), coagulation factor XIII A chain (F13A1), fibrinogen alpha chain (FIBA), fibrinogen beta chain (FIBB), and fibrinogen gamma chain (FIBG), were elevated for GBM samples (supplemental File S10), in comparison to the controls.

On comparison among GBM subtypes, cystatin B (CTSB) was elevated for PRO compared to MES and CLA subtype, and hemicentin-2 (HMCN2) and plexin-B1, which were increased and decreased for PRO *versus* CLA subtype, respectively (supplemental Fig. S4*B*). No proteins were observed to be significantly altered for the CLA *versus* MES subtype comparison.

**Fig. 3:**
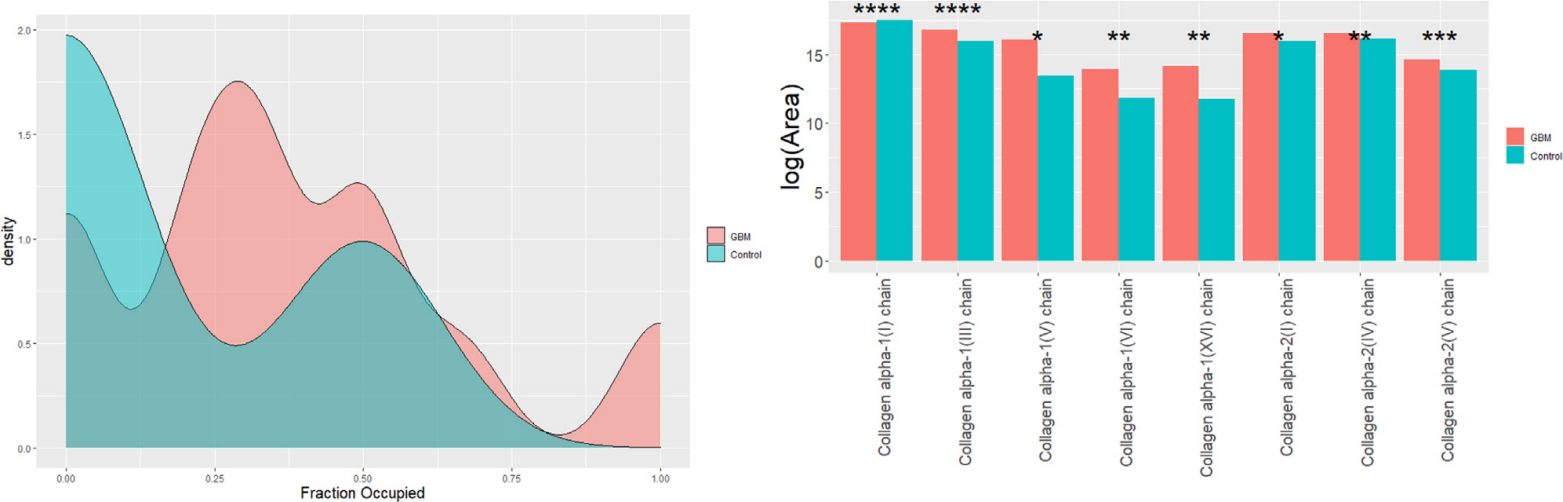
**Collagen hydroxyprolination.** Density plot showing proline hydroxylation of collagen peptides for GBM *versus* control (*left*). The x-axis shows the percentage of proline residues that are hydroxylated, and the y axis shows arbitrary units of density. The bar plots (*right*) show log-transformed normalized abundances for HPRO peptides for collagen proteins. Eight collagen proteins were significantly different in GBM *versus* control (∗*p* < 0.05, ∗∗*p* < 0.01, ∗∗∗*p* < 0.001, ∗∗∗∗*p* < 0.0001). The normalized log-transformed abundances were corrected for multiple comparisons. GBM, glioblastoma.

For peptide analysis (supplemental File S10), several *N*-deamidated and hydroxyprolinated (HPRO) peptides were increased in GBM *versus* control samples. Specifically, a number of HPRO peptides for collagens were found to be significantly elevated in GBM as compared to control samples. Several collagens were also increased at the protein level for GBM compared to the control samples. The degree of proline hydroxylation in collagen proteins was determined by dividing the number of hydroxylated proline residues by the total number of proline residues in a given peptide. A density plot was then generated showing the percentage of proline residues that are hydroxylated (Fig. 3). It was observed that control samples had far lesser HPRO peptide residues than GBM, which were mostly concentrated at 0% and a smaller cluster at 50%. In the GBM samples, the proline residues were commonly about 30% hydroxylated, but another hydroxylated cluster around 50% (the cluster for control at 50% was lower in density than GBM), and a smaller cluster at 100% was also observed. Further, we analyzed the differences in normalized abundances of HPRO peptides for various collagens observed in this study (supplemental File S11). Eight HPRO collagen proteins were significantly increased for GBM *versus* Control (Fig. 3). Among them, the collagen alpha-1(I) chain (CO1A1), collagen alpha-1(III) chain (CO1A3), and collagen alpha-2(IV) chain (CO4A2) were the top three most HPRO collagens. We then compared the abundances of each HPRO residue on these top-three collagens and observed a position-specific increase in HPRO in GBM *versus* Control (supplemental Fig. S5). Abundances for all HPRO residues for eight significantly HPRO collagens are provided in supplemental File S12. We then explored the matrisome data to investigate the IDH-mutant *versus* WT and EGFR amplified *versus* unamplified status of the samples. 30 proteins were observed to be significantly (FDR < 0.05) lower in abundance for mutant IDH1 compared to the WT. Notably, two PGs, CSPG4 and agrin were significantly lower about 6-fold for IDH-mutant compared to the WT (Fig. 2*B* and supplemental File S10). No significantly regulated proteins were observed for EGFR amplified *versus* unamplified samples.

### Gene Set Enrichment Analysis and Network Topology Analysis

The differentially regulated proteins (supplemental File S10) for GBM *versus* control were then analyzed by GSEA and NTA, which uses the TCGA RNAseq GBM database. Interestingly, various cancer-related pathways, including pathways in cancer, ECM–receptor interaction pathway, and PI3K-Akt signaling pathways, were among the enriched pathways (supplemental Fig. S6*A* and supplemental File S13). Among gene ontology (GO) analysis, cell-substrate junction and endoplasmic reticulum were the most enriched cellular components for control and GBM samples. For biological processes, positive regulation of defense response and negative regulation of cell development and for molecular function, organic acid-binding and sulfur (GAG) compound binding were among the most enriched for control and GBM sample, respectively (supplemental Fig. S6*A*). For NTA, WebGestalt uses a random walk method to compare the differentially regulated proteins in the present data with the TCGA RNAseq GBM database. The network for these along with enriched genes is shown ins. Using this analysis, we observed various enriched GO terms (red) in the network (supplemental Fig. S6*B*), including important cancer-related biological processes such as ECM organization (supplemental Fig. S6*C*), tumor development (supplemental Fig. S6*D*), and cell adhesion (supplemental Fig. S6*E*). To summarize, a number of cancer-related and GBM-related pathways and GO terms were observed enriched in our matrisome data, highlighting the connection of matrisome and GBM.

### Glycoproteomics Similarity Analysis

The raw data were searched using the publicly available in-house GlycReSoft user interface (desktop version) (27, 28), using a fasta file generated from UniProtKB (supplemental File S8) comprising of *N*- and *O*-glycosylated proteins and matrisomal proteins. GlycReSoft provided a list of 532 glycopeptides observed across the samples (supplemental File S9). The intensity of these glycopeptides was log-transformed and then analyzed using RAMZIS, an R toolkit for assessing glycoproteomic data using contextual similarity (38). For the analysis, a filter of glycopeptides present in at least two biological replicates was applied that yielded 198 (GBM *versus* control), 147 (Pro *versus* control), 91 (MES *versus* control), 136 (CLA *versus* control), 151 (CLA *versus* PRO), 121 (CLA *versus* MES), and 129 (PRO *versus* MES) glycopeptides for different comparisons performed. The null and test hypothesis ranking and internal distribution ranking with z-scores for various comparisons are provided in supplemental Files S14 and S15, respectively.

**Fig. 4:**
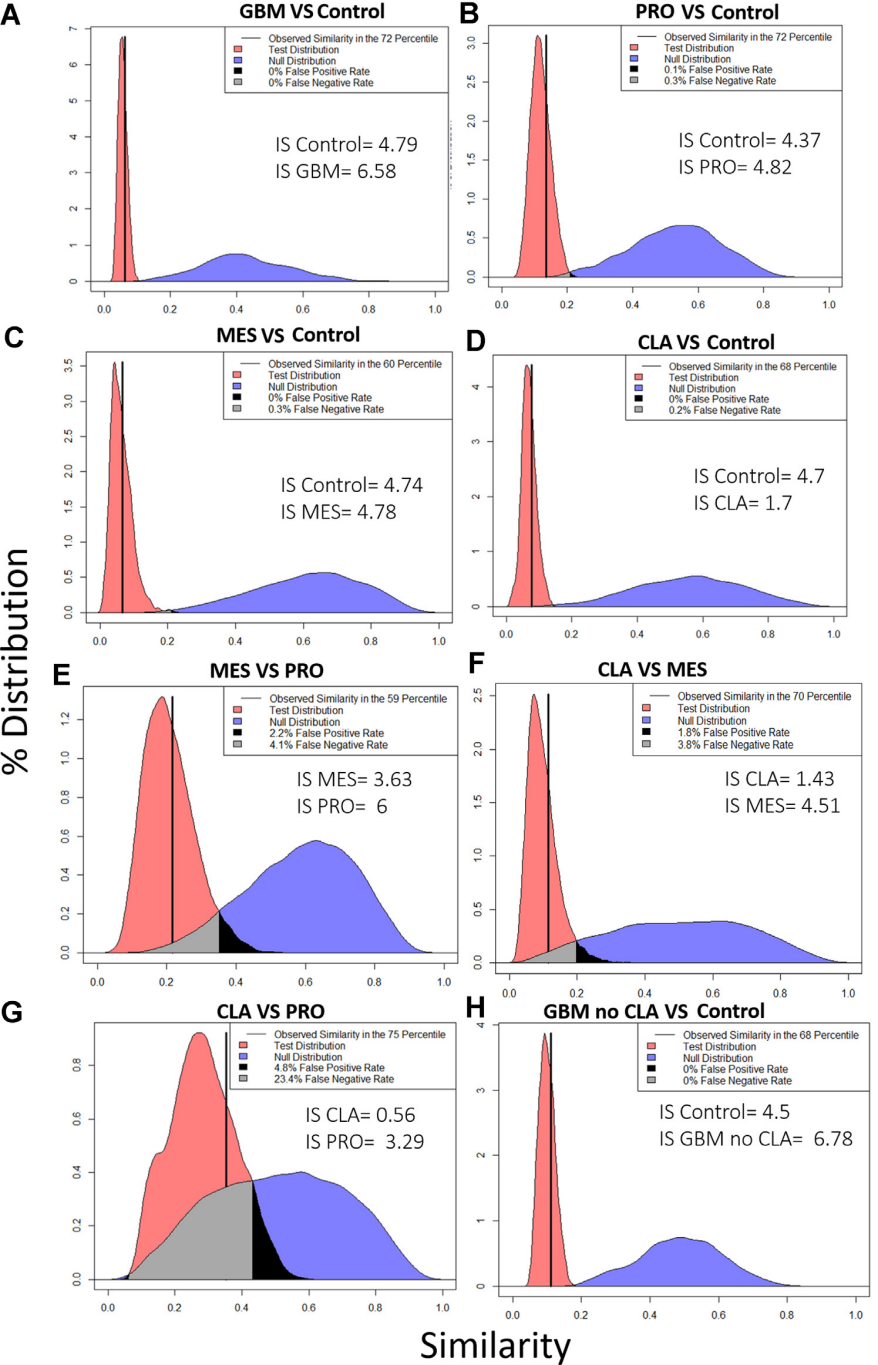
**Similarity plots using RAMZIS.** The modified Tanimoto similarity metric is represented on the x-axis, and the y-axis represents the percent of similarity distribution present at a given similarity. The observed similarity is represented by the solid *vertical black line*. As a metric of simulation functionality and reasonability, the observed similarity is placed within the test distribution as a percentile; an observed similarity within two standard deviations of the mean and within the two center quartiles passes the quality metrics. The test and null distributions are represented in *red* and *blue colors*, respectively. The *gray shaded region* within the comparisons represent the overlap of the two distributions that can be perceived as a false negative observation, wherein it is possible to observe a nontest similarity comparison but more likely for it to be a test similarity comparison; the *black shaded region* is considered false positives with a test observation possible, but a nontest observation being more likely. We accept up to a 20% false negative rate and a 5% false positive rate in line with other statistical standards. The observed similarity should be within the central quartiles (25%–75%) if RAMZIS has correctly simulated the data. Internal Similarity Confidence scores (IS) should be greater than 2, indicating a less than 3% chance that the internal and test similarity means could be conflated. Ideal similarity distributions are tightly defined with minimal tails; broader distributions indicate less reproducible similarity comparisons. *A*, GBM *versus* control. *B*, PRO *versus* control. *C*, MES *versus* control. *D*, CLA *versus* control; *E*, MES *versus* PRO. *F*, CLA *versus* MES. *G*, CLA *versus* PRO. *H*, GBM no CLA *versus* control. CLA, classical; GBM, glioblastoma; MES, mesenchymal; PRO, proneural.

For GBM *versus* control comparison, both internal similarity distributions (supplemental Fig. S7, *A* and *B*) had confidence scores above 2, indicating a less than 2.2% chance of the internal and test similarity distributions being equivalent and allowing us to reject the null hypothesis that they were equivalent. Both have negligible overlap between the internal and test. The internal similarity variance of control samples was equivalent to the other independent groups, which supported our conclusions despite the dependence within the control sample because the group variance was roughly equivalent to the sample variance. As a metric of simulation functionality and reasonability, the observed similarity was placed within the test distribution as a percentile; for GBM *versus* control similarity comparison (Fig. 4*A*), the observed similarity was in the 72nd percentile of the test distribution, making it well within two standard deviations of the mean and within the two center quartiles, passing the quality metrics (see Glycopeptide Similarity Analysis method section for details). There was negligible overlap between the test and null hypotheses, and we rejected the Null hypothesis that the test and null were equivalent; the glycosylation patterns were not being sampled from the same underlying distribution. The quality of the data and the separation allowed us to conclude the differences between the glycosylation patterns between the control and GBM samples (supplemental File S15).

The comparison of PRO *versus* control (supplemental Fig. S8, *A* and *B*) had high internal similarity with high confidence. We concluded from the RAMZIS similarity comparison of PRO *versus* control (Fig. 4*B*) that we could reject the null hypothesis; control and PRO did not sample from the same underlying distribution. Thus, control and PRO were reliably differentiated in glycosylation patterns. Similar profiles were observed for MES *versus* control (supplemental Fig. S8, *C* and *D*) and Figure 4*C*.

For the CLA *versus* control subtype, the internal similarity comparison remained confident for this comparison, but the internal of the CLA group score fell below the confidence threshold of 2 (supplemental Fig. S8, *E* and *F*). CLA also had a 3.4% false negative rate and a 0.2% false positive rate for the internal comparison. This indicated that members of the CLA subtype may have overlapped with the control group, and there was not a high enough degree of reproducibility in the CLA subtype to produce a reliable comparison. For the CLA *versus* control CLA similarity comparison (Fig. 4*D*), the observed similarity fell within acceptable bounds, but due to the CLA subtype's poor internal data quality, we could not draw a conclusion.

For the GBM *versus* control without the CLA subtype, both the internal similarities were sufficiently confident to draw conclusions with scores over 2 (supplemental Fig. S7, *C* and *D*). The observed similarity (Fig. 4*H*) was within the central quartiles and two standard deviations of the mean of the test similarity. The quality of the data and the separation indicated there were differences in the glycosylation patterns between the control and GBM subtypes. There was a slight change in the internal confidence scores of this comparison compared to the comparison between the control and all GBM subtypes; the decrease in the internal of the control likely stems from the increased similarity of the test comparison overall, as the control group itself is not different from the original comparison. The increase in the internal confidence of the GBM group stemmed from the absence of CLA, which must have less in common with PRO and MES than they did with one another or was much more variant than them; further comparisons suggest that CLA was much more variant. The increase in confidence was subtle but strong enough to work counter to the increased similarity in the test comparison.

For CLA *versus* MES (supplemental Fig. S9, *A* and *B* and Fig. 4*F*) and CLA *versus* PRO (supplemental Fig. S9, *C* and *D* and Fig. 4*G*) comparisons, CLA continued to have too poor data quality to indicate with confidence if there were any differences in glycosylation pattern in these comparisons, although general comparison showed evidence to reject the null hypothesis if there were stronger data for CLA subtype. The comparison of CLA *versus* PRO showed an increase in test similarity, indicating the two had more similar glycosylation patterns; due to CLA’s high internal variance and PRO’s lower degree of internal variance, as indicated by their internal similarity distributions, PRO may be a more well-regulated subset of the CLA subtype in regards to glycosylation. At the least, this suggested that the glycosylation of PRO had more in common with CLA than any other experimental group examined here. This was further confirmed in MES *versus* PRO (supplemental Fig. S9*E* and *F*, and Fig. 4*E*), where both MES and PRO had high internal confidences, an observed similarity in the central quartiles of the test distribution and false-positive and negative rates under our thresholds to reject the null hypothesis. Thus, MES and PRO were reliably differentiated in glycosylation patterns.

### Glycoproteomics Analysis

Based on the results of similarity analysis, we filtered ranked glycopeptides based on dissimilarity for the group comparisons using z scores cutoff of −1 (supplemental File S16). Further, we selected only those glycopeptides which were observed in both the groups (highlighted; as indicated by TRUE; NA means not observed in a group) to remove any false positive difference and provide reliable differentiable patterns and glycopeptides with high confidence. Using these criteria, we found 17 glycopeptides dissimilar between different group comparisons, including GBM *versus* control, GBM (no CLA) *versus* control, PRO *versus* control, MES *versus* control, CLA *versus* control, MES *versus* PRO, CLA *versus* PRO, and CLA *versus* MES. Notably, many of these glycopeptides belonged to PGs, including BCAN (S625, T590), VCAN (T937, T939), and AGRN (T1335). Interestingly, most of the glycopeptides were reduced in GBM (with or without CLA) or GBM subtypes, except sialylated *O*-glycopeptide at site T590, which was higher for both GBM (no CLA) and PRO subtype *versus* control samples, indicating the elevation of sialylation in GBM. In addition, *N*-glycopeptides for neuronal cell adhesion molecule at sites N908 and N996 were also reduced for GBM (no CLA), MES, and CLA subtypes *versus* controls. Furthermore, two *O*-glycopeptide at T937 and T939 for VCAN were elevated for MES *versus* PRO subtype (Table 4). On the other hand, *O*-glycopeptide at T1335 for AGRN was elevated for PRO *versus* MES. CLA subtype showed lower glycopeptide intensity compared to MES and PRO subtype, which could also be attributed to poor quality data, as explained in the previous section. However, one sialylated *N*-glycopeptide (N129) belonging to fibrinogen gamma showed a 10-fold higher intensity for MES than the CLA subtype. The tandem mass spectra for all these glycopeptides are shown in Supplemental File S17.

**Table 4:** Filtered ranked glycopeptides based on dissimilarity for the group comparisons using z scores cutoff of −1 and observed in both the groups, their associated proteins, average intensities for Group 1 and Group 2, and direction of change for Group 1 *versus* 2 (increased or decreased in Group 1)

Comparison Group 1 *versus* Group 2	Glycopeptide	Protein	Average intensity Group 1	Average intensity Group 2	Direction of change for Group 1 *versus* Group 2
GBM *versus* Control	EVGEATGGPELS(O-Glycosylation)GVPR{Hex:1; HexNAc:1; Neu5Ac:1}	Q96GW7|PGCB_HUMAN Brevican core protein	4149179.809	13700681.18	↓
GBM *versus* Control	VHGPPT(O-Glycosylation)ETLPTPR{Hex:2; HexNAc:2}	Q96GW7|PGCB_HUMAN Brevican core protein	6489435.887	14198380.17	↓
GBM (no CLA) *versus* Control	VHGPPT(O-Glycosylation)ETLPTPR{Hex:2; HexNAc:2; Neu5Ac:1}	Q96GW7|PGCB_HUMAN Brevican core protein	17032540.09	1732376.149	↑
GBM (no CLA) *versus* Control	VNVVN(N-Glycosylation)STLAEVHWDPVPLK{Hex:5; HexNAc:2}	Q92823|NRCAM_HUMAN Neuronal cell adhesion molecule	746852.5772	2514727.657	↓
PRO *versus* Control	VHGPPT(O-Glycosylation)ETLPTPR{Hex:2; HexNAc:2; Neu5Ac:1}	Q96GW7|PGCB_HUMAN Brevican core protein	24156172.47	1732376.149	↑
PRO *versus* Control	GSLSYLN(N-Glycosylation)VTR{Hex:5; HexNAc:2}	P07339|CATD_HUMAN Cathepsin D	1188954.931	11732037.79	↓
PRO *versus* Control	GSLSYLN(N-Glycosylation)VTR{Hex:6; HexNAc:2} }	P07339|CATD_HUMAN Cathepsin D	2380641.715	14259589.44	↓
MES *versus* Control	VNVVN(N-Glycosylation)STLAEVHWDPVPLK{Hex:5; HexNAc:2}	Q92823|NRCAM_HUMAN Neuronal cell adhesion molecule	2091187.216	2514727.657	↓
CLA *versus* Control	FN(N-Glycosylation)HTQTIQQK{Hex:5; HexNAc:2}	Q92823|NRCAM_HUMAN Neuronal cell adhesion molecule	2962900.074	36541412.68	↓
CLA *versus* Control	LLNINPN(N-Glycosylation)KT{Hex:5; HexNAc:2}	P11279|LAMP1_HUMAN Lysosome-associated membrane glycoprotein 1	1271305.87	10969186.96	↓
MES *versus* PRO	FQPTTST(O-Glycosylation)GIAEK{Hex:4; HexNAc:4}	P13611|CSPG2_HUMAN Versican core protein	2504868.943	2780108.302	↓
MES *versus* PRO	FQPTT(O-Glycosylation)STGIAEK{Hex:4; HexNAc:4}	P13611|CSPG2_HUMAN Versican core protein	2504868.94	2780108.3023	↓
MES *versus* PRO	LPSSAVT(O-Glycosylation)PR{HexNAc:2}	O00468|AGRIN_HUMAN Agrin	9163338.025	3284955.444	↑
CLA *versus* PRO	LLNINPN(N-Glycosylation)K{Hex:5; HexNAc:2}	P11279|LAMP1_HUMAN Lysosome-associated membrane glycoprotein 1	4037102.859	1165363.714	↑
CLA *versus* PRO	TFVLSALQPSPTHSS(O-Glycosylation)SNTQR{Hex:1; HexNAc:1; Neu5Ac:1}	P19827|ITIH1_HUMAN Inter-alpha-trypsin inhibitor heavy chain H1	2836091.638	1161018.844	↑
CLA *versus* MES	EEQFNS(O-Glycosylation)TFR{Fuc:1; Hex:4; HexNAc:4}	P01859|IGHG2_HUMAN Immunoglobulin heavy constant gamma 2	50183304.3	13546469.66	↑
CLA *versus* MES	DLQSLEDILHQVEN(N-Glycosylation)K{Hex:5; HexNAc:4; Neu5Ac:1}	P02679|FIBG_HUMAN Fibrinogen gamma chain	180503261.1	15778062.01	↑

Abbreviations: CLA, classical; GBM, glioblastoma; MES, mesenchymal; PRO, proneural.

We further investigated the expression of glycosylation enzymes (glycosyltransferase/glycosidase) in GBM and GBM subtype *versus* control samples (Fig. 2*C* and supplemental File S10). Strikingly, a significant increase in glycosylation enzymes (FDR < 0.05) was observed in GBM *versus* controls (Fig. 2*C*). Specifically, dolichyl-diphosphooligosaccharide–protein glycosyltransferase subunit STT3A and dolichyl-diphosphooligosaccharide–protein glycosyltransferase subunit STT3B were about 120- and 43-fold increased for GBM *versus* controls, indicating higher glycosylation in GBM. A similar increase in STT3A was observed for PRO subtype *versus* control samples. In addition, other glycosyltransferases, including beta-1 3-glucosyltransferase, mannose-1-phosphate guanylyltransferase beta, and dolichyl-diphosphooligosaccharide–protein glycosyltransferase subunit one and two were also increased for GBM *versus* control samples (supplemental File S10). Notably, glycosidase enzymes including mannosyl-oligosaccharide glucosidase, glucosidase two subunit beta, and neutral alpha-glucosidase AB were elevated for GBM samples; specifically, PRO and MES subtypes compared to control samples (supplemental File S10), indicating an increase in maturation and processing of high mannose *N*-glycans to complex *N*-glycans in GBM.

## Discussion

Altered protein and gene expression in GBM using proteomics and genomic techniques are well-documented, but glycomics and glycoproteomics investigation in GBM is underexplored. This study provided an in-depth glycomics, proteomics, and glycoproteomics analysis of GBM subtypes and control samples with a special focus on matrisome. The aim was to achieve a comprehensive understanding of matrisome molecules, including PGs and GAGs in GBM tumorigenesis, to better understand the disease pathogenesis and identify clinical markers.

For the study, we utilized our state-of-the-art *on-slide* tissue digestion method and extracted various GAGs (CS and HS disaccharides) followed by peptides using appropriate enzymes from formalin-fixed paraffin-embedded TMAs. The tumors were assigned GBM subtypes based on genome patterns: 11 CLA, nine MES, and 19 PRO. Additionally, four control tissue samples were used, making a total of 43 samples.

We first analyzed the CS and HS disaccharides extracted from GBM and control samples. CS and HS GAGs may directly promote tumor cell adhesion, proliferation, and invasion, causing a more aggressive form of brain cancer (41, 42). Moreover, changes in GAG sulfation patterns play an essential role in physiological processes in various diseases, including cancer and neurological disorders (43). Importantly, aberrant accumulation of CS has been seen in ECM glioma (44). Chondroitin sulfate synthase 1, CS polymerization enzyme, has been significantly upregulated in GBM compared to normal brain tissue, and chondroitin sulfate synthase 1 mediates the increase of 6-*O* sulfation of CS in human glioma (44), supporting our finding of elevated 6-*O* CS sulfation in GBM samples. In addition, both 4-*O* sulfation and 6-*O* sulfation have been observed in human glioma cells that could be used as a possible target in glioma therapy (45). We observed increased unsulfated CS and HS disaccharides for GBM compared to normal samples. An increase in extracellular sulfatase (SULF1 and SULF2) in human GBM regulates important interactions of growth factors and ECM components to PGs, thus mediating tumor cell invasion (46) and proliferation (47). The sulfatase enzyme selectively cleaves HS 6-*O* sulfation, thus indicating a lower HS 6-*O* sulfation in GBM as observed in this study (41, 46). Congruent with our observation, an increase in CS 6-*O* sulfation, increase in HS unsulfation, and decrease in HS 6-*O* sulfation was observed in breast cancer (48). These changes in the HS sulfation pattern result in high heparanase expression which in turn degrades the ECM and facilitates metastasis (43, 48). We also observed specific GAG disaccharide features for GBM subtypes which have not been reported before; however, a previous study has reported differences in SULF1 and SULF2 enzyme level between GBM subtypes (10). Thus, our findings in concordance with previous studies point toward the therapeutic potential of GAGs in GBM.

We further analyzed the matrisome protein and peptide expression. Several core matrisome-related proteins were observed among differentially expressed proteins, majority of them increased, while only five were reduced in GBM, including TNR, HAPLN1, HAPLN2, DCN, and LAMB2. TNR has been observed to decrease for highly malignant cancer (49). On the contrary, the increased expression of DCN has been reported in GBM tumors negatively associated with the overall survival rate of GBM patients (50). Reduction in HAPLNs in malignant gliomas has been previously observed. CSPGs in the normal brain interact with tenascins, HAPLNs, and other glycoproteins to form matrix scaffolds and ECM aggregates around subsets of neurons, known as perineuronal nets, which are associated with reduced neuronal cell motility and synaptic plasticity (51). But, in malignant gliomas, CSPGs are not associated with HAPLNs and thus may contribute to changes in the matrix scaffold and perineuronal nets affecting ECM solubility and promoting the invasion of gliomas cells to neural tissue (52). A tumor and metastasis protein LGI1 was reduced in GBM samples; this is consistent with significant reduction, inactivation, or absence of LGI1 observed in malignant gliomas, making it a strong tumor suppressor gene candidate involved in the malignant progression (53). The top-most abundant protein for GBM was VWA1, which was about 100-fold higher for PRO and CLA subtype *versus* controls. No significant difference in VWA1 was observed for IDH1 mutant *versus* WT samples. Interestingly, plasma von Willebrand factor has been reported to increase in GBM *versus* controls and suggested as a circulating biomarker of disease malignancy (53).

Intriguingly, a majority of matrisomal proteins were increased for GBM compared to control samples, including various collagens, laminins, fibronectin, tenascins, fibrinogens, and various PGs, including CSPG4, AGRN, BGN, glypican 1, and PRELP. CSPG4 has been suggested as a biomarker for GBM that facilitates tumorigenesis and tumor progression (20, 54). Multiple HSPGs and CSPGs core proteins and related enzymes were upregulated in GBM tumors relative to normal brain, including various genes previously implicated in tumor invasion and tumorigenesis and observed by the present study, including glypican 1, CSPG4, BGN, and AGRN (10). In contrast, PRELP was observed to be reduced in other cancers (55, 56). The collagens, laminins, and fibronectin constitute fibrous proteins in the brain matrisome (56). The increase in these fibrous proteins may increase the fibrous network in the brain, thus promoting GBM cell adhesion, invasion, and migration (57). Additionally, these proteins influence the behavior of glioma cells and have been detected within the basal lamina of tumor blood vessels (58). These results clearly indicate the involvement of the ECM in the aggressive and malignant nature of GBM (58).

We also showed that the degree or percentage of collagen peptide HPRO was also different between GBM and control tissue, the latter having lower HPRO peptide residues than GBM. Collagen biogenesis requires collagen prolyl 4-hydroxylase (P4H) activity to catalyze collagen proline hydroxylation. Increased mRNA levels of P4HA1 and P4HA2 have been observed in human breast cancer facilitating collagen deposition and further promoting invasion and metastasis (59). Additional work will be required to understand the effect of the increased collagen HPRO in GBM.

Several blood coagulation-related proteins were increased for GBM *versus* control samples, including VWA1, ANT3, F13A1, FIBA, FIBB, and FIBG. Interestingly, malignant diseases such as cancer have been associated with hypercoagulation, and several coagulation markers have been associated with brain tumor growth, progression, and metastasis, indicating an association of tumor and thromboembolic risk (60).

We further investigated the altered matrisome proteins in the IDH1 mutant *versus* WT status of the samples. Thirty proteins were decreased for IDH1 mutant compared to WT, including CSPG4 and AGRN as the least abundant proteins. Conversely, CSPG4 has been previously reported to increase for both EGFR and PDGFRA amplified tumors (10). Additionally, CSPG4 (also known as NG2) has been reported previously to be frequently expressed in both IDH-mutant and WT GBM, but not lower grade astrocytomas pointing toward its prognostic and therapeutic relevance as a tumor-associated antigen for antibody-based immunotherapy (61).

Aberrant glycosylation is strongly associated with cancer progression and plays fundamental roles in numerous steps of cancer development, such as interfering with the cell–cell adhesion, cell–ECM interaction, stimulating the RTK signaling pathway, and promoting angiogenesis (53). Elevated expression of lecticans, namely VCAN (CSPG2), NCAN, and BCAN, have been reported in GBM (46). While we did not see any significant elevation at the protein level, we observed several significantly altered *O*-glycopeptides, including several peptides for VCAN elevated in GBM. In contrast, *O*-glycopeptides have been reported to reduce for NCAN compared to controls (62, 63). Additionally, overexpression of NCAN protein has been reported in neuroblastoma (62). Importantly, sialylated O-glycopeptide for BCAN at site T590 was higher for both GBM (no CLA) and PRO subtype *versus* control samples, while sialylated *O*-glycopeptide at S625 was increased for GBM *versus* control samples. Notably, sialylated glycans have been recently reported to be elevated in canine glioma (64). An increase in BCAN in GBM has been linked to increased invasiveness, and sialylation in cancer is related to adhesion and invasion mechanisms increasing metastatic potential of cancer cells (65, 66). These reports, together with our findings, suggest the involvement of glycosylation in GBM tumorigenesis.

Moreover, a peptide and two *O*-glycopeptides in the GAG (alpha) binding region for VCAN were elevated for MES *versus* PRO subtype, while *O*-glycopeptide for AGRN and *N*-glycopeptide for fibrinogen was increased for PRO and CLA subtype *versus* MES, indicating differential *N*-and *O*-glycosylation at subtype level. Interestingly, *N*-glycopeptide for neuronal cell adhesion molecule, a neural adhesion protein, was decreased for GBM compared to controls. Historically, it has been observed that altered glycosylation mediates a decrease in cancer cell adhesion and increases ECM interaction that may contribute to the invasive and metastatic potential of cancer cells (67, 68). Additionally, a sialylated *N*-glycopeptide (N129) for fibrinogen gamma showed a 10-fold higher intensity for MES than the CLA subtype. Notably, both fibrinogen and sialylation have been indicated to be involved in cancer metastasis (69, 70), pointing toward a probability of higher invasive potential of MES compared to the CLA subtype.

To correlate the glycoproteomics findings, we scrutinized the proteomics data to investigate glycosyltransferase and glycosidase enzyme expression in GBM *versus* control samples. Intriguingly, we observed an increase in both glycosyltransferase and glycosidase enzymes in GBM *versus* controls, specifically, dolichyl-diphosphooligosaccharide-protein glycosyltransferase various subunit (STT3A, STT3B, 1, 2, 48 kDa). These are catalytic subunits of the oligosaccharyltransferase complex that catalyzes the first step in protein *N*-glycosylation that is the initial transfer of Glc3Man9GlcNAc2 from the dolichol-PP lipid precursor to an asparagine residue on a peptide in a consensus sequon Asn-X-Ser/Thr (71). Thus, indicating an increase in *N*-glycosylation for GBM *versus* control. In addition, various mannosidase and glucosidase that cleaves mannose and glucose residues from Glc2Man9GlcNAc2 oligosaccharide precursors catalyzing the processing and maturation of high mannose type *N*-glycans to more complex type *N*-glycans (71) were also elevated for GBM *versus* control, indicating increased *N*-glycan processing in GBM. A high expression of highly matured multiantennary fucosylated and sialylated N-glycans has been observed in high-grade glioma cells associated with malignant cell behaviors (72).

The similarity analysis of glycopeptide also clearly indicated reliable differentiable glycosylation patterns between GBM *versus* control, PRO *versus* control, MES *versus* control, and PRO *versus* MES comparisons. However, the analysis revealed some heterogeneity in the glycoproteomics data of the CLA subtype, which could be attributed to missing values or a high degree of internal variance. Alternatively, CLA might be too broadly defined as a subtype or its subtype phenotype may be characterized by highly unregulated glycosylation. The present glycoproteomics analysis was performed without any enrichment; thus, in the future, a comprehensive glycoproteomics analysis with enrichment techniques should be employed for a better understanding of the role of ECM glycosylation in GBM.

We also performed GSEA and NTA analysis on the differentially regulated matrisome proteins and observed sulfur (GAG) compound binding as the most enriched molecular function for the GBM samples, again emphasizing the importance of GAG, its sulfation pattern, and binding to matrisome molecules in GBM. Moreover, we performed network topology analysis on our data using TCGA RNAseq GBM data and found common genes between the two studies belonging to various crucial networks, including matrisome organization, cell adhesion, and tumor development, all of which have been reported to be enriched in GBM (22, 73) and that promote matrisome-mediated glioma invasion, metastasis, and tumorigenesis.

**Fig. 5:**
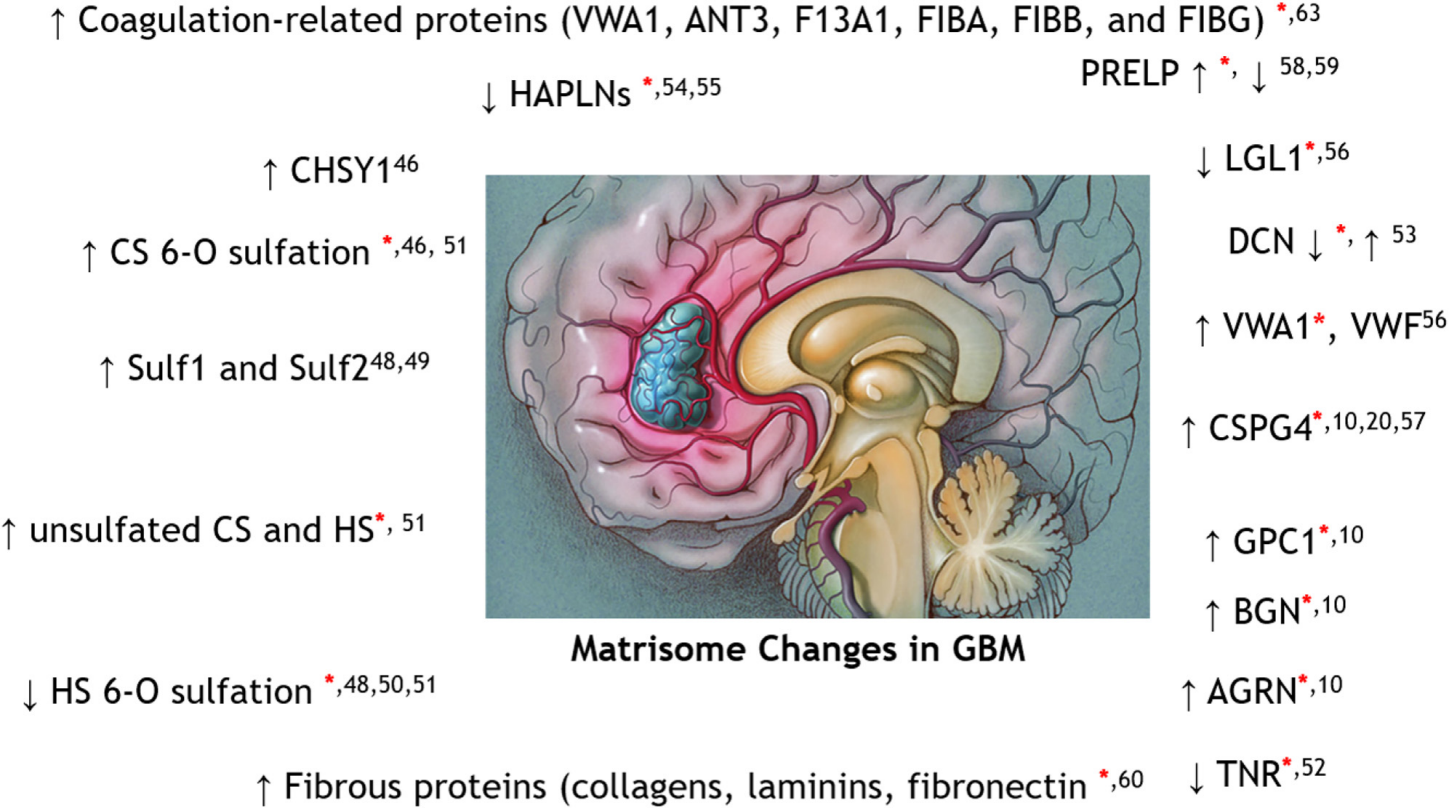
**A summary of key matrisome component changes in GBM as observed by this study (*red asterisk* ∗) congruent with the literature (number indicate the references to the literature)**.

In summary, this study confirmed not only previous data and findings but also produced novel findings for a better understanding of GBM pathogenesis. Fig.5 summarizes changes in the key matrisome component in GBM as observed by this study congruent with literature. However, these findings were based on relatively small sample sets, particularly for controls that were from a single patient and did not have independence from one another; thus, these findings need to be verified with larger cohorts as the next step. In addition, glycoproteins could be enriched to achieve better glycopeptide coverage. Finally, this study identified alterations in key matrisomal molecules, the knowledge of which will enable the exploitation of novel markers and therapeutic targets for GBM and inform the role of glycosylation and ECM in GBM.

## Data Availability

The mass spectral data files have been deposited to the ProteomeXchange Consortium (http://proteomecentral.proteomexchange.org) *via* the PRIDE partner repository (74, 75) with the dataset identifier PXD028931. The peaks project file is available at Zenodo with 10.5281/zenodo.5911810.

## Supplemental data

This article contains supplemental data.

## Conflict of interest

Authors declare no conflict of interest.
