# Supplementary material for: In-Depth Matrisome and Glycoproteomic Analysis of Human Brain Glioblastoma Versus Control Tissue
Source: Mol Cell Proteomics. 2022 Feb 23;21(4):100216. doi: 10.1016/j.mcpro.2022.100216 (PMC8957055; doi:10.1016/j.mcpro.2022.100216)

1. sp|Q96GW7|PGCB_HUMAN Brevican core protein
EVGEATGGPELS(O-Glycosylation)GVPR{Hex:1; HexNAc:1; Neu5Ac:1} (S625)

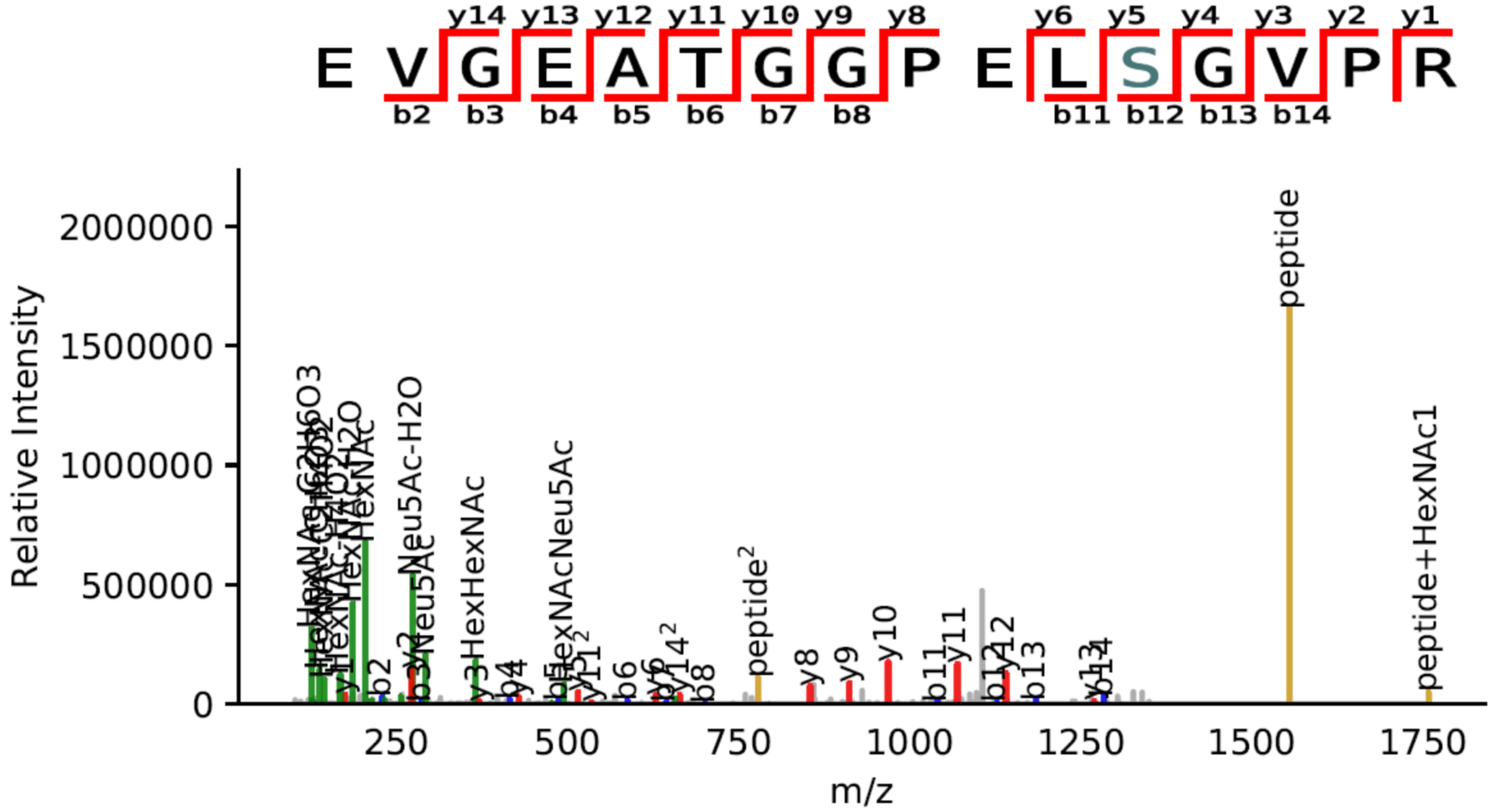

2. sp|Q96GW7|PGCB_HUMAN Brevican core protein
VHGPPT(O-Glycosylation)ETLPTPR{Hex:2; HexNAc:2} (T590)

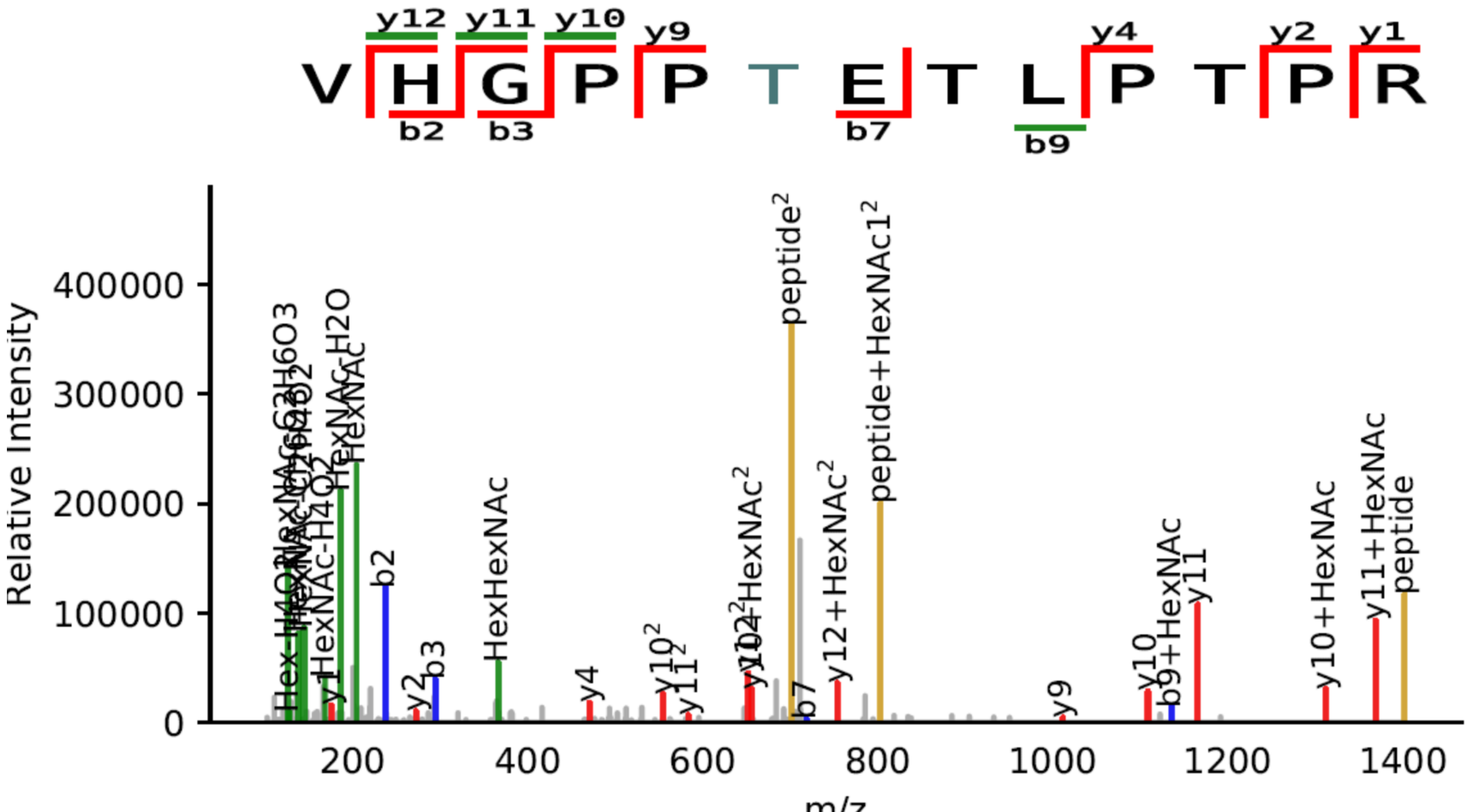

3&5 sp|Q96GW7|PGCB_HUMAN Brevican core protein

VHGPPT(O-Glycosylation)ETLPTPR{Hex:2; HexNAc:2; Neu5Ac:1}(T590)

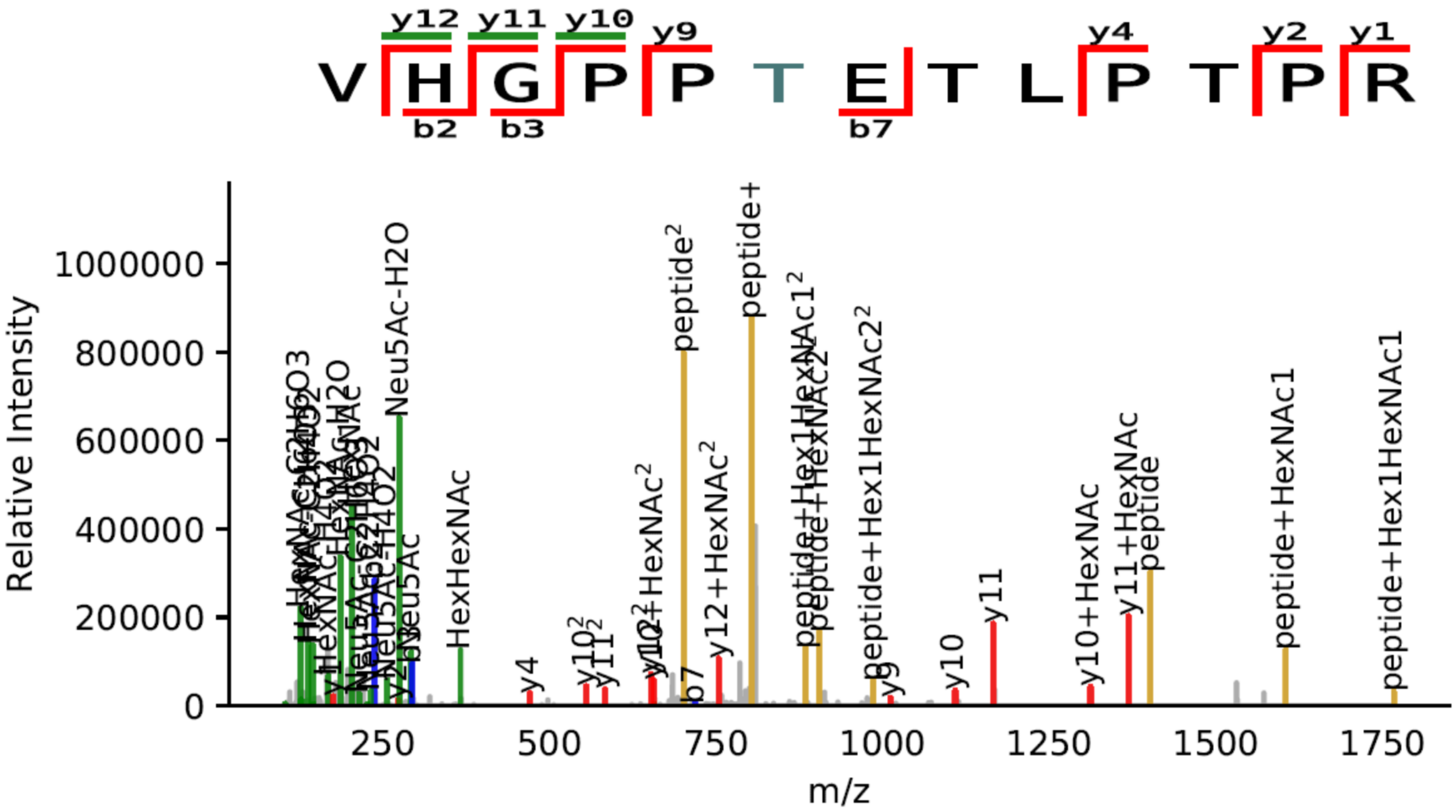

4&8. sp|Q92823|NRCAM_HUMAN Neuronal cell adhesion molecule OS=Homo sapiens OX=9606 GN=NRCAM PE=1 SV=3
VNVVN(N-Glycosylation)STLAEVHWDPVPLK{Hex:5; HexNAc:2}(N908)

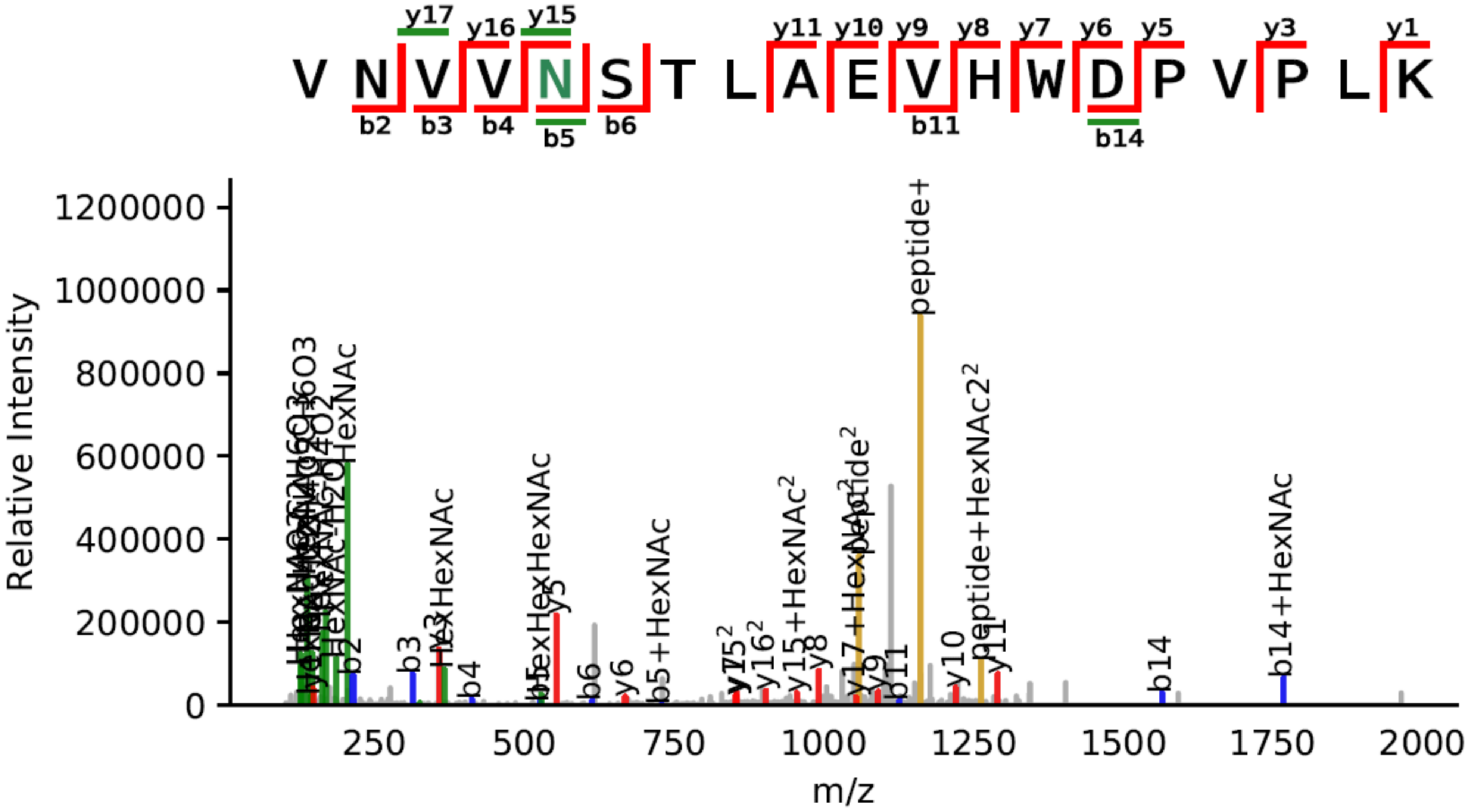

6. sp|P07339|CATD_HUMAN Cathepsin D OS=Homo sapiens OX=9606 GN=CTSD PE=1 SV=1
GSLSYLN(N-Glycosylation)VTR{Hex:5; HexNAc:2} (N313)

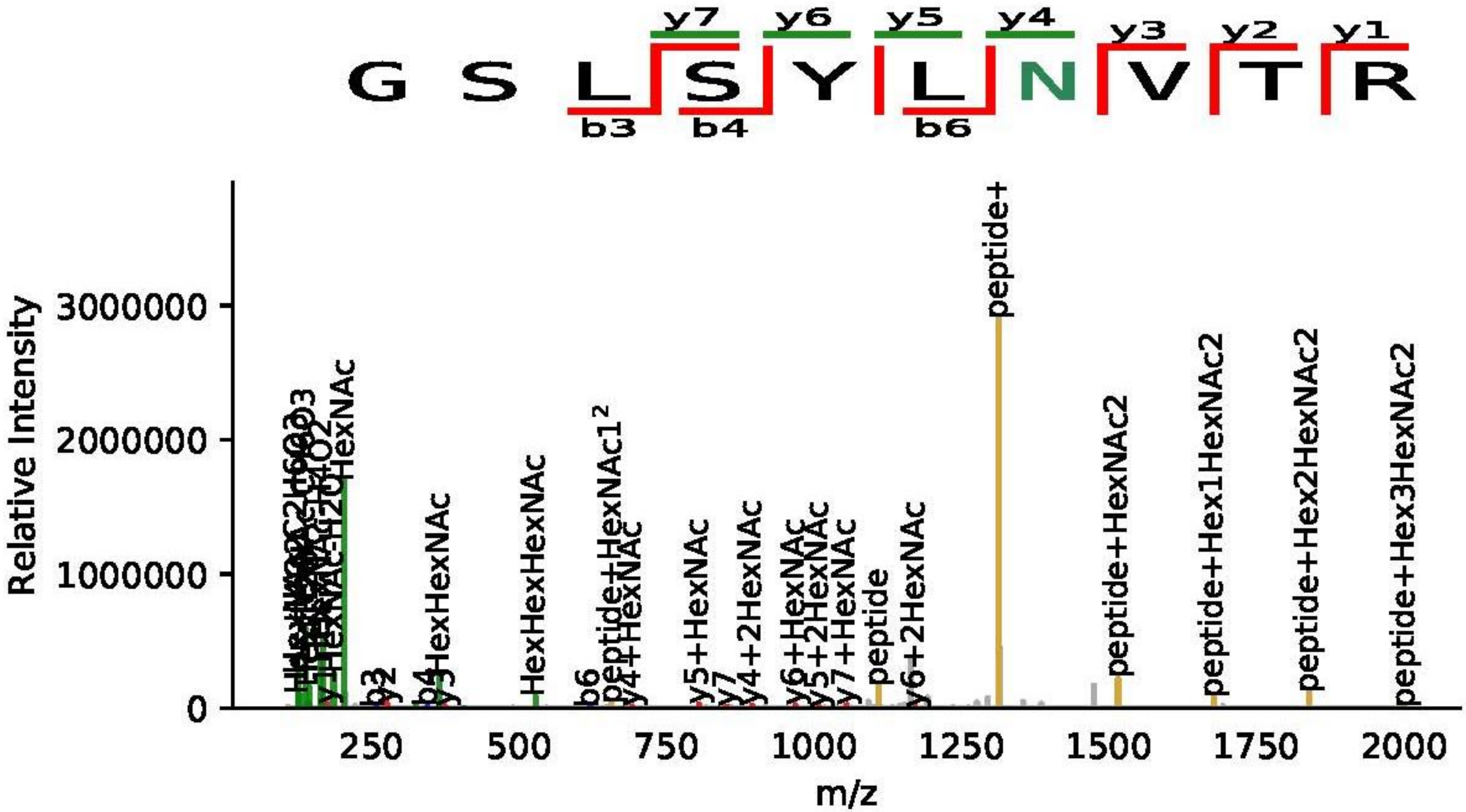

7. sp|P07339|CATD_HUMAN Cathepsin D OS=Homo sapiens OX=9606 GN=CTSD PE=1 SV=1
GSLSYLN(N-Glycosylation)VTR{Hex:6; HexNAc:2} (N313)

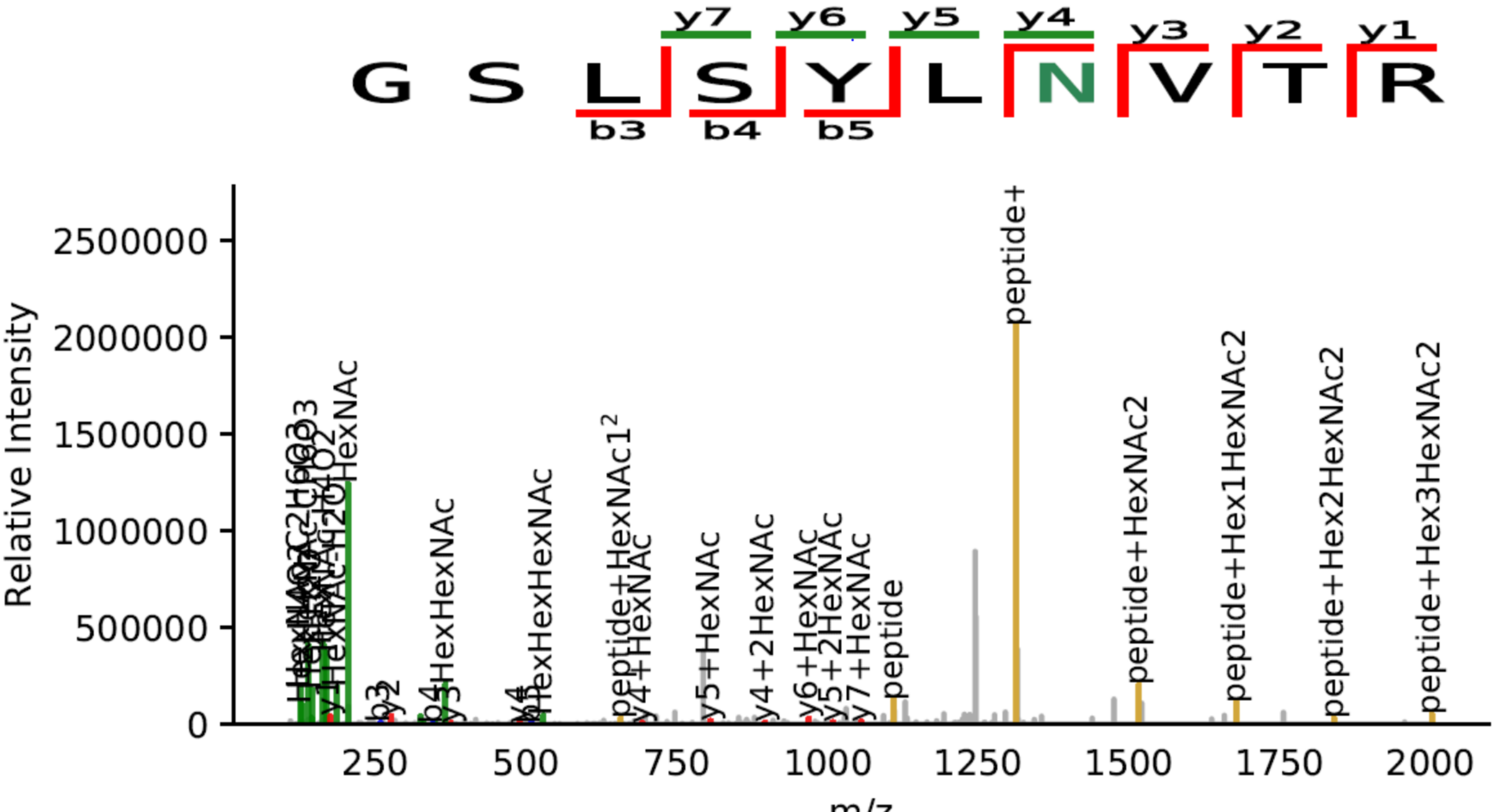

9. sp|Q92823|NRCAM_HUMAN Neuronal cell adhesion molecule OS=Homo sapiens OX=9606 GN=NRCAM PE=1 SV=3

FN(N-Glycosylation)HTQTIQQK{Hex:5; HexNAc:2} (N996)

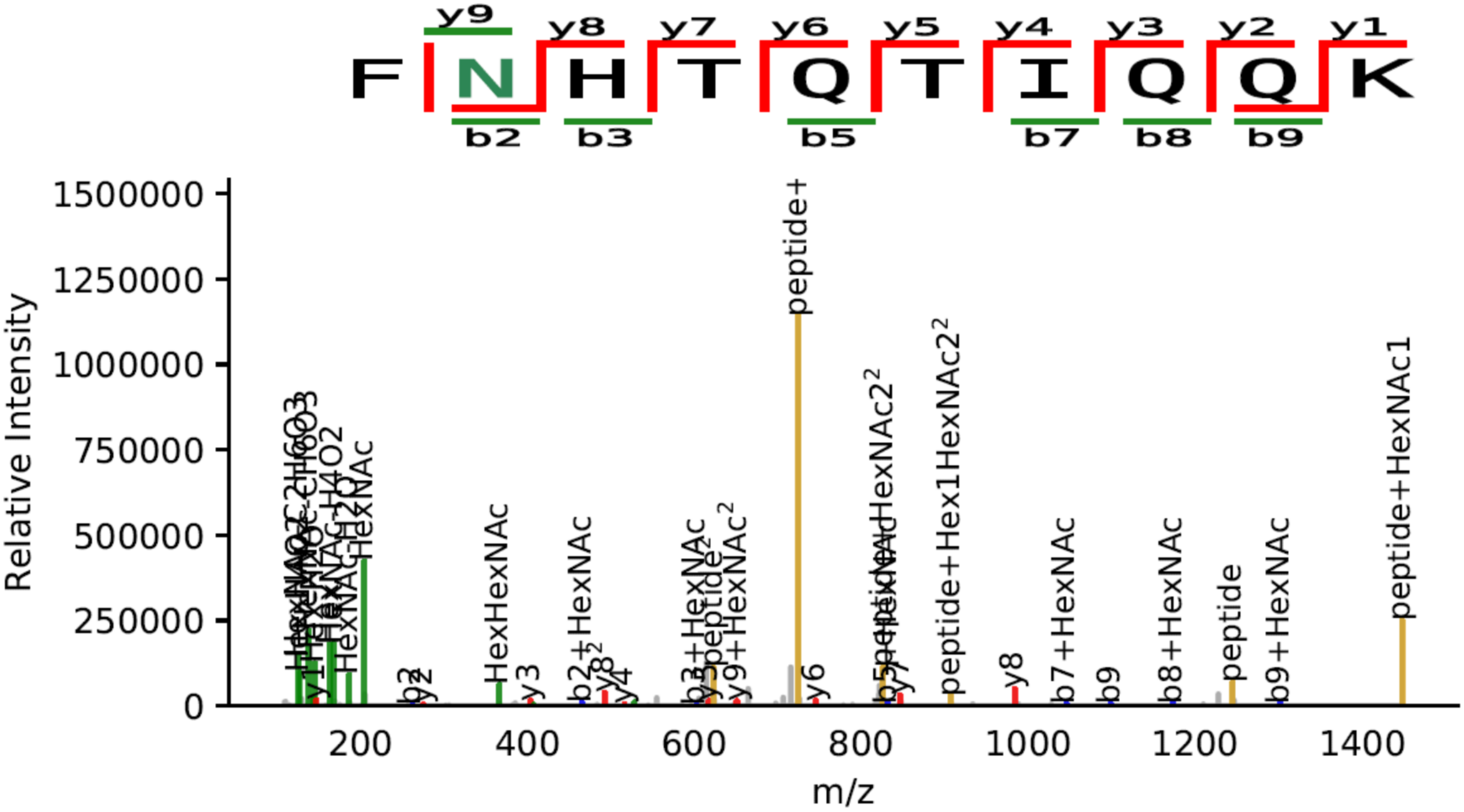

10 and 14. sp|P11279|LAMP1_HUMAN Lysosome-associated membrane glycoprotein 1 OS=Homo sapiens OX=9606 GN=LAMP1 PE=1 SV=3

LLNINPN(N-Glycosylation)KT{Hex:5; HexNAc:2} (N311)

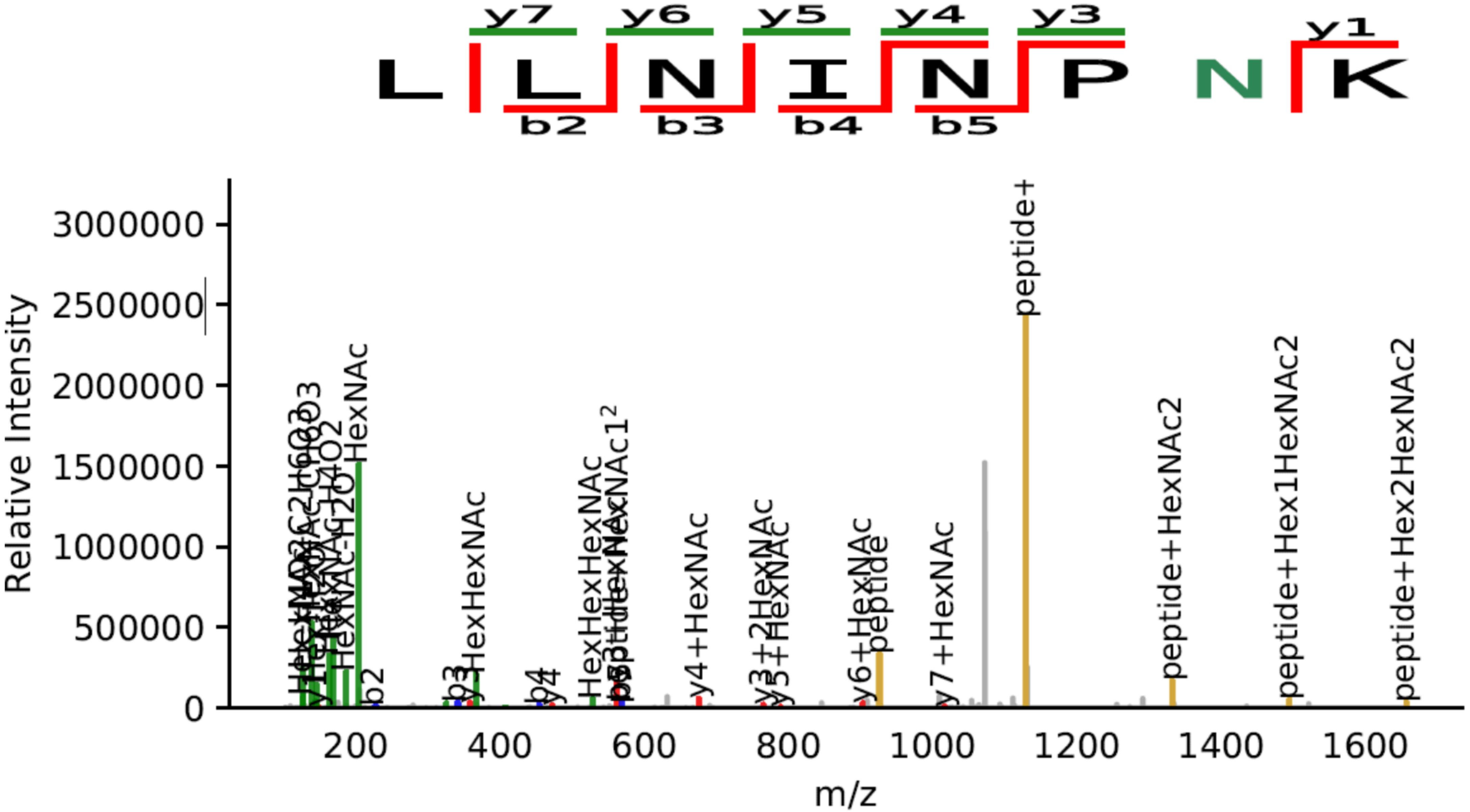

11. sp|P13611|CSPG2_HUMAN Versican core protein OS=Homo sapiens OX=9606 GN=VCAN PE=1 SV=3
FQPTTST(O-Glycosylation)GIAEK{Hex:4; HexNAc:4} (T939)

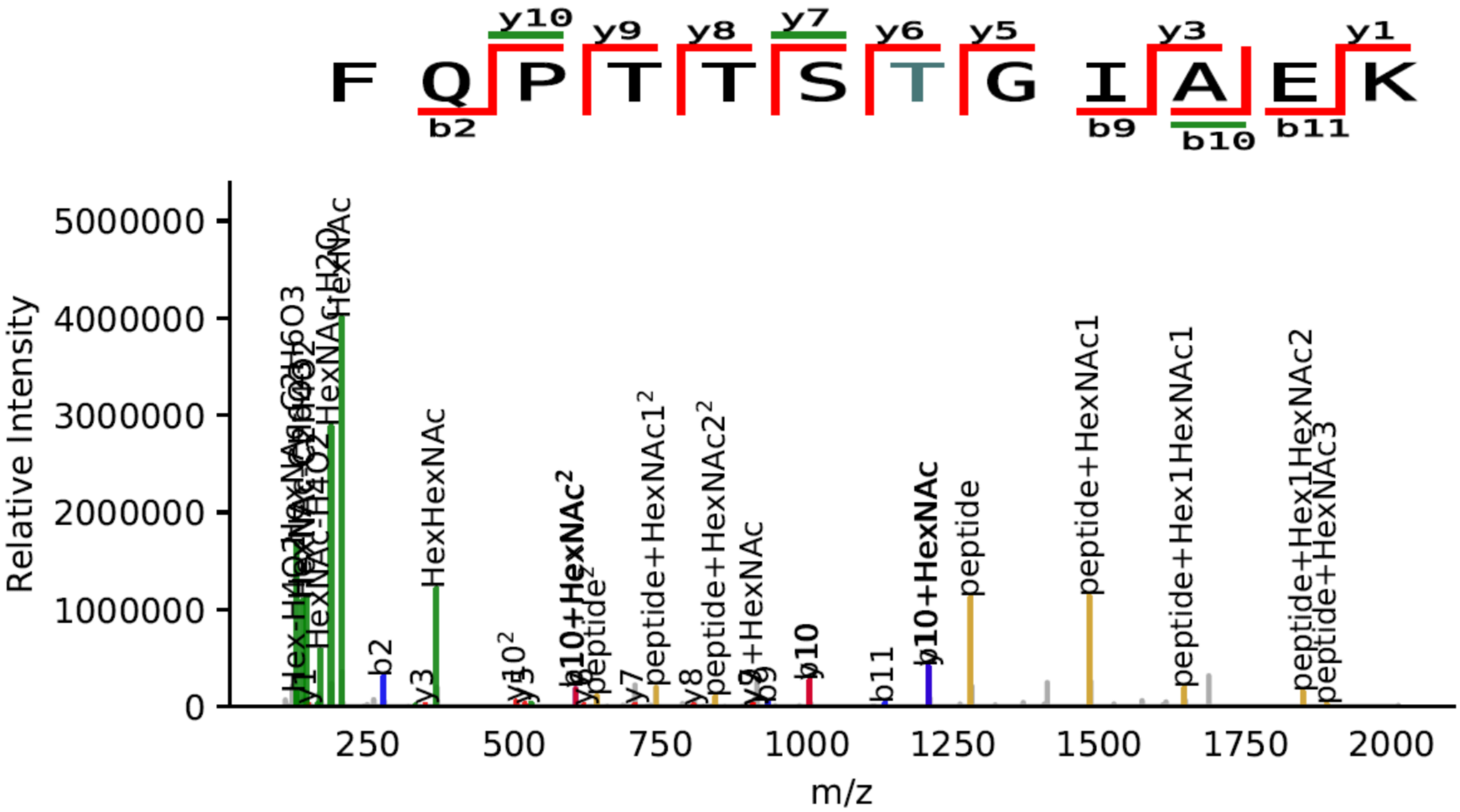

12. sp|P13611|CSPG2_HUMAN Versican core protein OS=Homo sapiens OX=9606 GN=VCAN PE=1 SV=3
FQPTT(O-Glycosylation)STGIAEK{Hex:4; HexNAc:4} (T937)

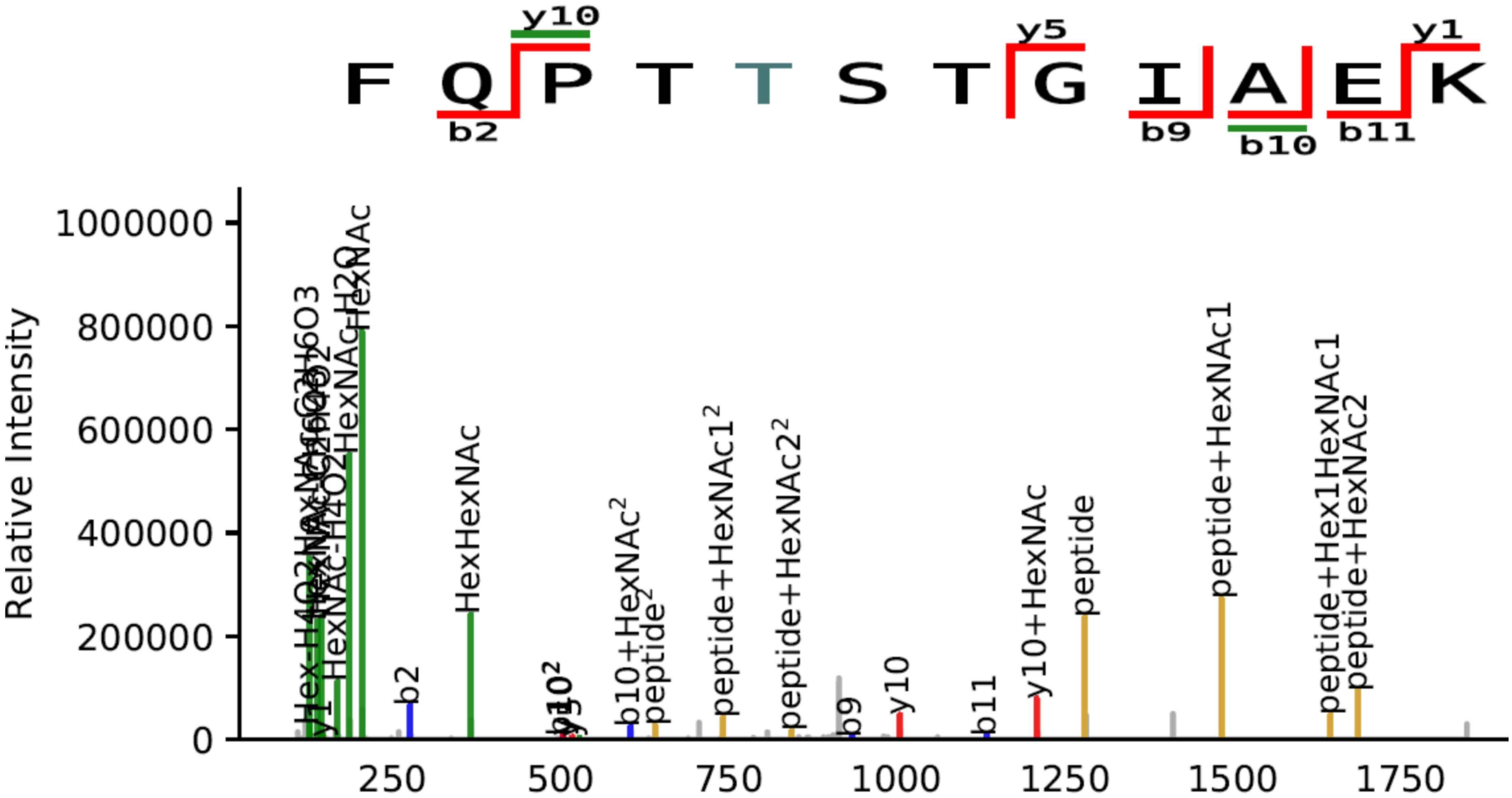

13. sp|O00468|AGRIN_HUMAN Agrin OS=Homo sapiens OX=9606 GN=AGRN PE=1 SV=6
LPSSAVT(O-Glycosylation)PR{HexNAc:2} (T1335)

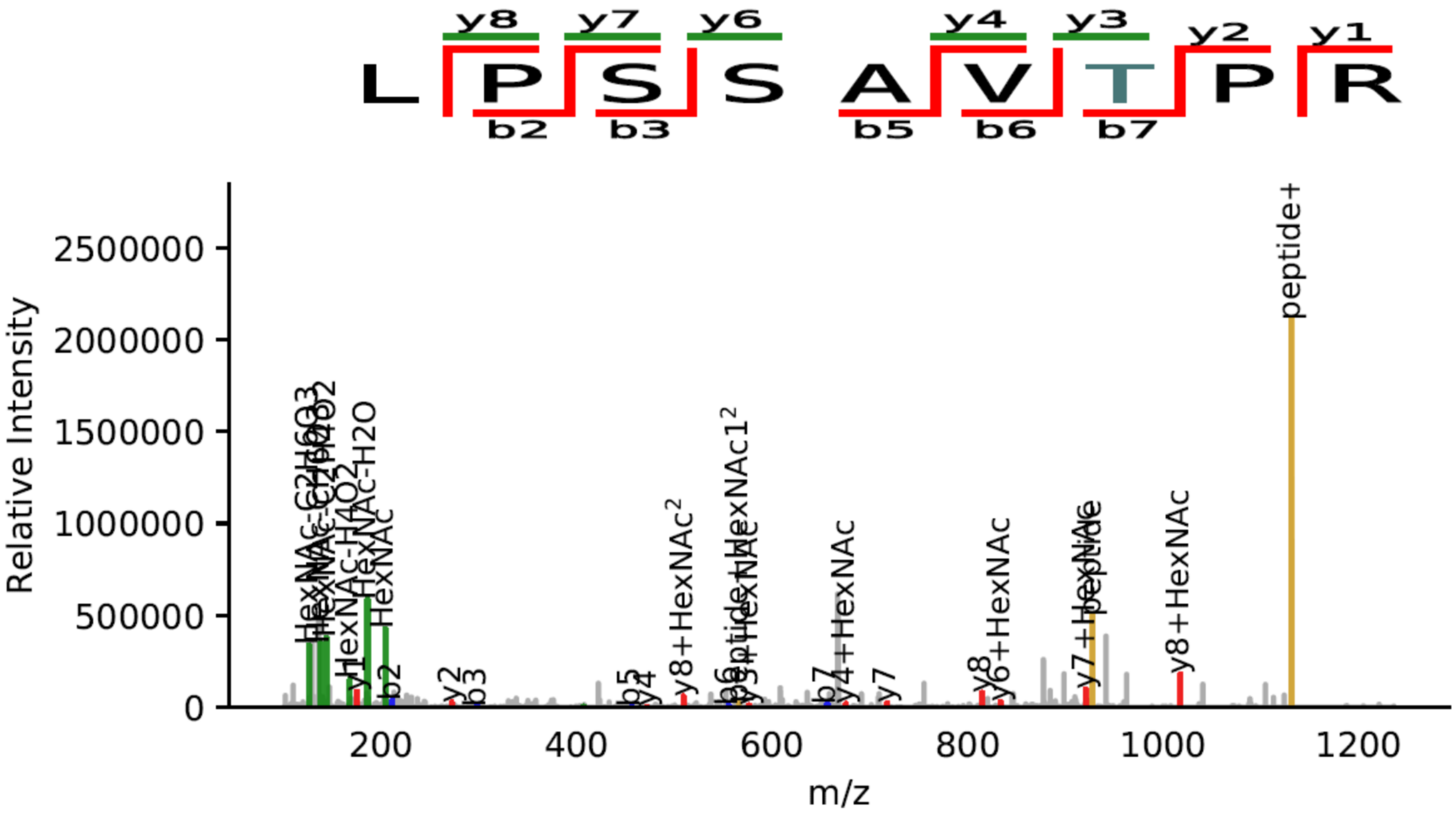

15. sp|P19827|ITIH1_HUMAN Inter-alpha-trypsin inhibitor heavy chain H1 OS=Homo sapiens OX=9606 GN=ITIH1 PE=1 SV=3

TFVLSALQPSPTHSS(O-Glycosylation)SNTQR{Hex:1; HexNAc:1; Neu5Ac:1} (S706)

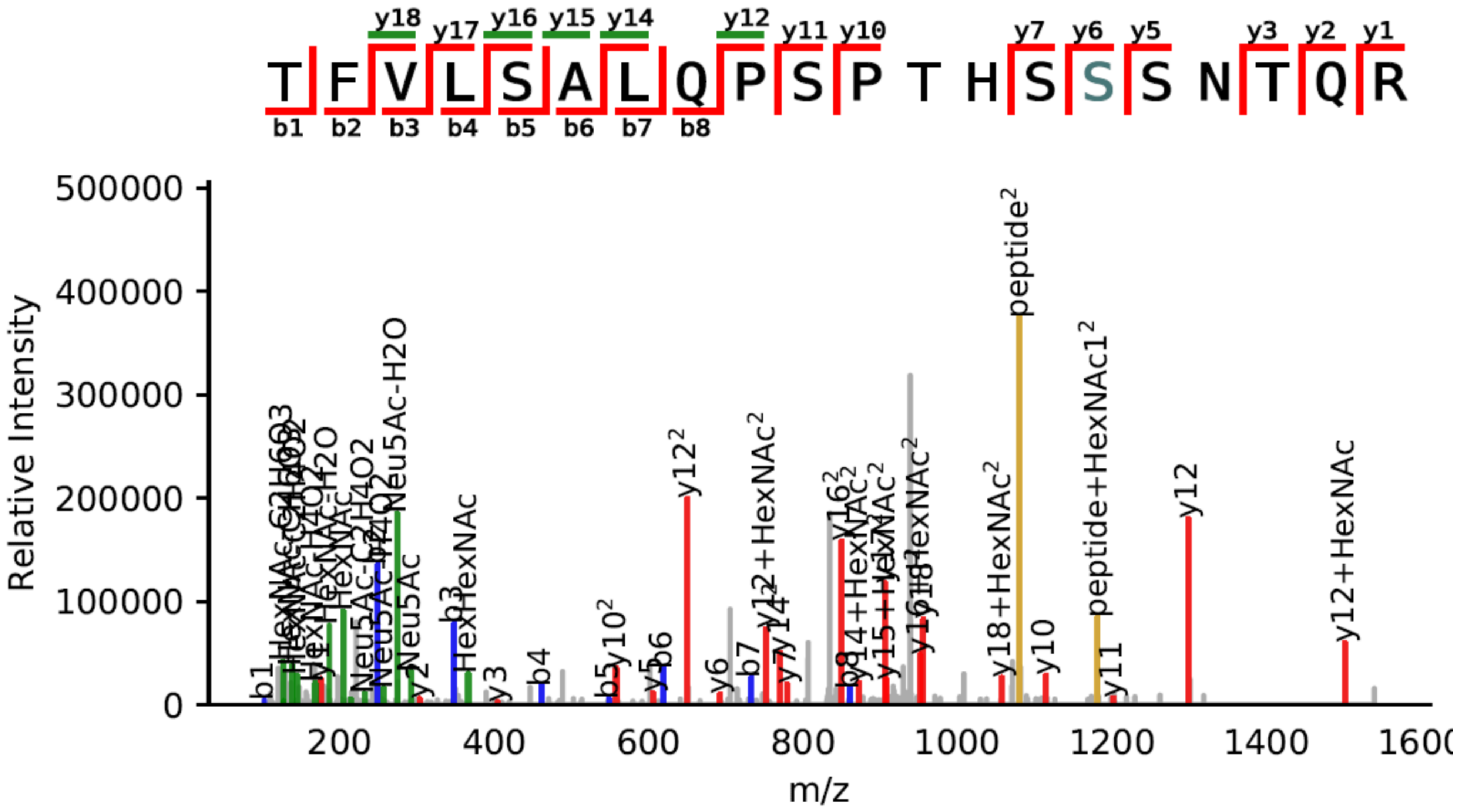

16. sp|P01859|IGHG2_HUMAN Immunoglobulin heavy constant gamma 2 OS=Homo sapiens OX=9606 GN=IGHG2 PE=1 SV=2

EEQFNS(O-Glycosylation)TFR{Fuc:1; Hex:4; HexNAc:4} (S227)

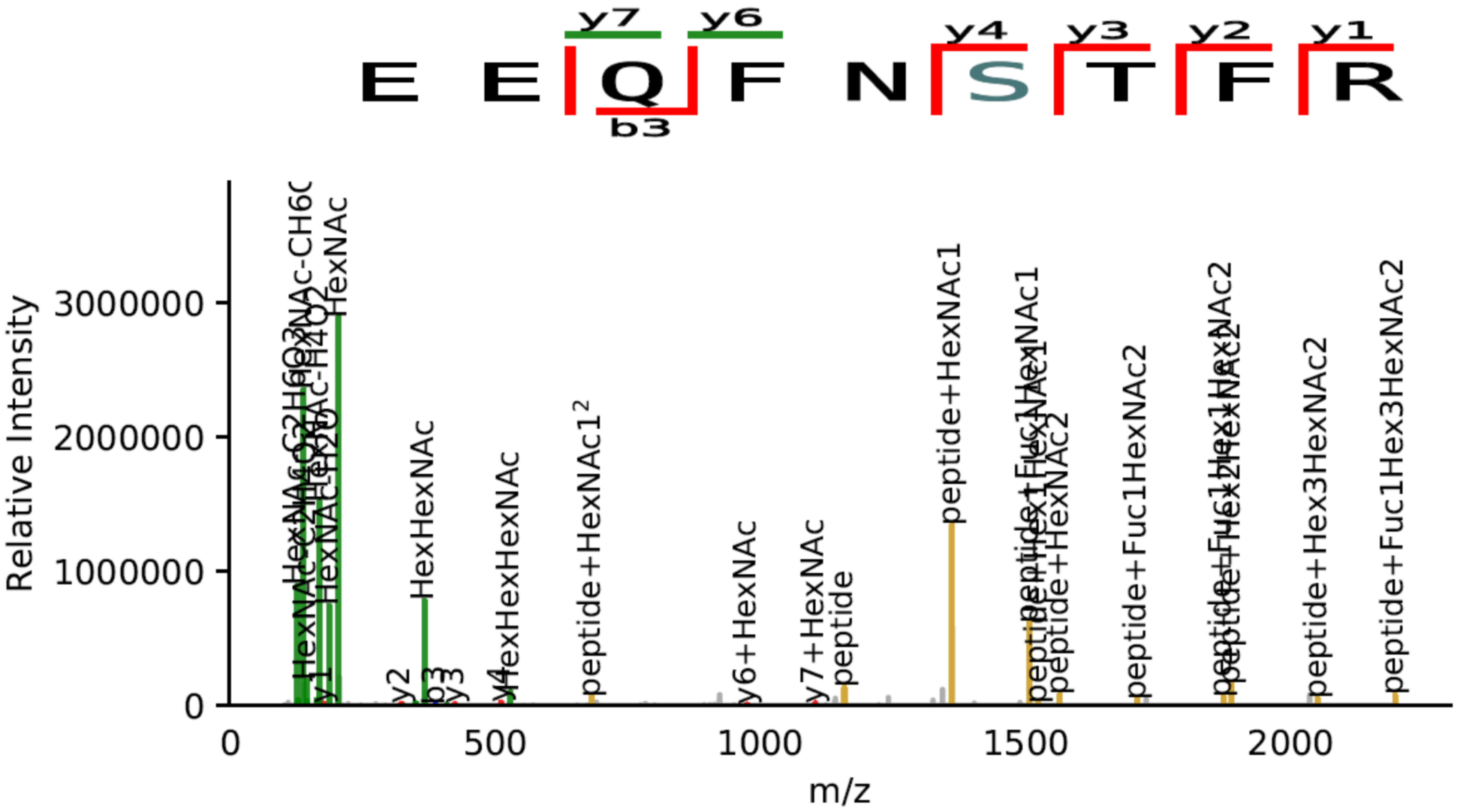

17. sp|P02679|FIBG_HUMAN Fibrinogen gamma chain OS=Homo sapiens OX=9606 GN=FGG PE=1 SV=3
DLQSLEDILHQVEN(N-Glycosylation)K{Hex:5; HexNAc:4; Neu5Ac:1}N129

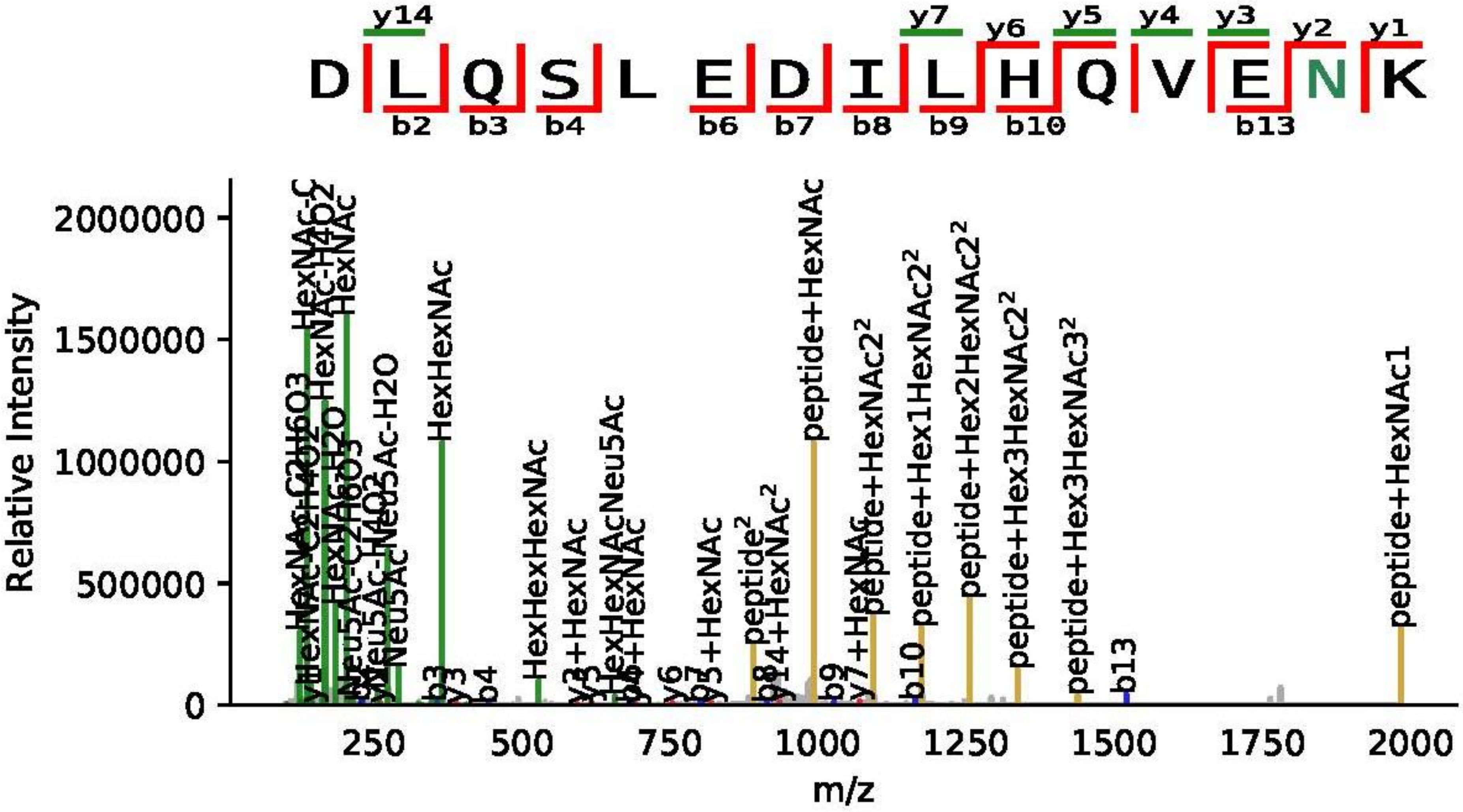

Supplement: Supplemental File S17 [file mmc17.pdf]
