# Supplementary material for: In-Depth Matrisome and Glycoproteomic Analysis of Human Brain Glioblastoma Versus Control Tissue
Source: Mol Cell Proteomics. 2022 Feb 23;21(4):100216. doi: 10.1016/j.mcpro.2022.100216 (PMC8957055; doi:10.1016/j.mcpro.2022.100216)
Supplement: Supplemental Tables S1–S2 and Figures S1–S9 [file mmc18.docx]

**In-depth matrisome and glycoproteomic analysis of human brain glioblastoma versus control tissue**

Manveen K. Sethi^1^, Margaret Downs^1^, Chun Shao^1^, William E. Hackett^1, 2^, Joanna J. Phillips^3,4^, Joseph Zaia^1, 2*^

^1^Dept. of Biochemistry, Center for Biomedical Mass Spectrometry, Boston University

^2^Bioinformatics Program, Boston University

^3^Dept. of Neurological Surgery, Brain Tumor Center, Helen Diller Family Cancer Research Center, University of California San Francisco

^4^Division of Neuropathology, Department of Pathology, University of California San Francisco

Corresponding author*

**Supplemental tables and figures-**

Supplemental Table S1. Additional sample information, including raw file names and peak database search numbers for proteomics analysis.

| **Coordinate** | **Disease Category** | **Expression Subtype** | **EGFR** | **IDH1** | **Raw File Name** | **PEAKS database search number** | **Slide Number** | **UCSF_Code** |
| --- | --- | --- | --- | --- | --- | --- | --- | --- |
| 2C4 | GBM | PRO | unamplified | mutant | 02_UCSFslide10_2C4 with 350fmol pepmix_120min_3.5ul injection_7491_10_2C4 | 1 | 10 | 07491 |
| 3D6 | GBM | PRO | unknown | wt | 03_UCSFslide8_3D6 with 350fmol pepmix_120min_3.5ul injection_8345_8_3D6 | 2 | 8 | 08345 |
| 3C2 | GBM | MES | unamplified | wt | 04_UCSFslide9_3C2 with 350fmol pepmix_120min_3.5ul injection_8858_9_3C2 | 3 | 9 | 08858 |
| 2D5 | GBM | MES | unknown | wt | 05_UCSFslide10_2D5 with 350fmol pepmix_120min_3.5ul injection_6969_10_2D5 | 4 | 10 | 06969 |
| 2A2 | Control | Cortex | unknown | wt | 06_UCSFslide10_2A2 with 350fmol pepmix_120min_3.5ul injection_S89-13028 control cortex_10_2A2 | 5 | 10 | Control 2A |
| 3B4 | GBM | PRO | unknown | mutant | 07_UCSFslide9_3B4 with 350fmol pepmix_120min_3.5ul injection_8354_9_3B4 | 6 | 9 | 08354 |
| 3A4 | GBM | CLA | unamplified | mutant | 08_UCSFslide8_3A4 with 350fmol pepmix_120min_3.5ul injection_6884_8_3A4 | 7 | 8 | 06884 |
| 3E | GBM | CLA | amplified | wt | 09_UCSFslide9_3E4 with 350fmol pepmix_120min_3.5ul injection_8061_9_3E4 | 8 | 9 | 08061 |
| 2E5 | Control | White  Matter | unknown | wt | 11_UCSFslide8_2E5 with 350fmol pepmix_120min_3.5ul injection_S89-13028 control white matter_8_2E5 | 9 | 8 | Control 1A |
| 2C2 | Control | Cortex | unknown | wt | 12_UCSFslide10_2C2 with 350fmol pepmix_120min_3.5ul injection_S89-13028 control cortex_10_2C2 | 10 | 10 | Control 2B |
| 3A1 | Control | WhiteMatter | unknown | wt | 13_UCSFslide9_3A1 with 350fmol pepmix_120min_3.5ul injection_S89-13028 control white matter_9_3A1 | 11 | 9 | Control 1B |
| 2B6 | GBM | PRO | unamplified | wt | 14_UCSFslide10_2B6 with 350fmol pepmix_120min_3.5ul injection_8386_10_2B6 | 12 | 10 | 8386 |
| 2C6 | GBM | CLA | amplified | wt | 15_UCSFslide10_2C6 with 350fmol pepmix_120min_3.5ul injection_7880_10_2C6 | 13 | 10 | 07880 |
| 3C5 | GBM | MES | unknown | wt | 16_UCSFslide8_3C5 with 350fmol pepmix_120min_3.5ul injection_8257_8_3C5 | 14 | 8 | 08257 |
| 3C4 | GBM | CLA | unknown | wt | 18_UCSFslide8_3C4 with 350fmol pepmix_120min_3.5ul injection_8216_8_3C4 | 15 | 8 | 08216 |
| 3B1 | GBM | PRO | unknown | wt | 19_UCSFslide9_3B1 with 350fmol pepmix_120min_3.5ul injection_8351_9_3B1 | 16 | 9 | 08351 |
| 3C1 | GBM | MES | unamplified | wt | 21_UCSFslide9_3C1 with 350fmol pepmix_120min_3.5ul injection_8858_9_3C1 | 17 | 9 | 08858 |
| 3D3 | GBM | MES | amplified | wt | 22_UCSFslide10_3D3 with 350fmol pepmix_120min_3.5ul injection_7686_10_3D3 | 18 | 10 | 07686 |
| 3D3 | GBM | PRO | unknown | wt | 23_UCSFslide8_3D3 with 350fmol pepmix_120min_3.5ul injection_8318_8_3D3 | 19 | 8 | 08318 |
| 2B1 | GBM | PRO | unamplified | mutant | 24_UCSFslide10_2B1 with 350fmol pepmix_120min_3.5ul injection_7884_10_2B1 | 20 | 10 | 07884 |
| 3B3 | GBM | MES | unamplified | wt | 25_UCSFslide8_3B3 with 350fmol pepmix_120min_3.5ul injection_7499_8_3B3 | 21 | 8 | 7499 |
| 3D2 | GBM | CLA | unknown | wt | 26_UCSFslide9_3D2 with 350fmol pepmix_120min_3.5ul injection_9418_9_3D2 | 22 | 9 | 9418 |
| 3D4 | GBM | PRO | unknown | wt | 27_UCSFslide8_3D4 with 350fmol pepmix_120min_3.5ul injection_8318_8_3D4 | 23 | 8 | 08318 |
| 3D4 | GBM | MES | amplified | wt | 28_UCSFslide10_3D4 with 350fmol pepmix_120min_3.5ul injection_7686_10_3D4 | 24 | 10 | 07686 |
| 2D2 | GBM | CLA | amplified | wt | 29_UCSFslide10_2D2 with 350fmol pepmix_120min_3.5ul injection_7034_10_2D2 | 25 | 10 | 07034 |
| 2B4 | GBM | PRO | unamplified | wt | 31_Lost some spray in the middle_UCSFslide10_2B4 with 350fmol pepmix_120min_3.5ul injection_9259_10_2B4 | 26 | 10 | 09259 |
| 2B2 | GBM | PRO | unamplified | mutant | 32_UCSFslide10_2B2 with 350fmol pepmix_120min_3.5ul injection_7884_10_2B2 | 27 | 10 | 07884 |
| 2D1 | GBM | PRO | unknown | wt | 33_UCSFslide8_2D1 with 350fmol pepmix_120min_3.5ul injection_8306_8_2D1 | 28 | 8 | 08306 |
| 3C3 | GBM | CLA | unknown | wt | 34_UCSFslide8_3C3 with 350fmol pepmix_120min_3.5ul injection_8216_8_3C3 | 29 | 8 | 08216 |
| 2B5 | GBM | PRO | unamplified | wt | 35_UCSFslide10_2B5 with 350fmol pepmix_120min_3.5ul injection_8386_10_2B5 | 30 | 10 | 8386 |
| 3A3 | GBM | CLA | unamplified | mutant | 36_UCSFslide8_3A3 with 350fmol pepmix_120min_3.5ul injection_6884_8_3A3 | 31 | 8 | 06884 |
| 3E5 | GBM | CLA | amplified | wt | 37_UCSFslide9_3E5 with 350fmol pepmix_120min_3.5ul injection_9459_9_3E5 | 32 | 9 | 9459 |
| 3D4 | GBM | CLA | amplified | wt | 41_UCSFslide9_3D4 with 350fmol pepmix_120min_3.5ul injection_9295_9_3D4 | 33 | 9 | 9295 |
| 2C3 | GBM | PRO | unamplified | mutant | 42_UCSFslide10_2C3 with 350fmol pepmix_120min_3.5ul injection_7491_10_2C3 | 34 | 10 | 7491 |
| 3C3 | GBM | MES | amplified | wt | 43_UCSFslide9_3C3 with 350fmol pepmix_120min_3.5ul injection_11-13797_9_3C3 | 35 | 9 | 5705 |
| 2D1 | GBM | CLA | amplified | wt | 44_UCSFslide10_2D1 with 350fmol pepmix_120min_3.5ul injection_7034_10_2D1 | 36 | 10 | 07034 |
| 3B6 | GBM | PRO | unamplified | wt | 45_UCSFslide9_3B6 with 350fmol pepmix_120min_3.5ul injection_8624_9_3B6 | 37 | 9 | 8624 |
| 3D2 | GBM | PRO | unknown | wt | 46_UCSFslide8_3D2 with 350fmol pepmix_120min_3.5ul injection_8306_8_3D2 | 38 | 8 | 08306 |
| 3B2 | GBM | PRO | unk | wt | 47_UCSFslide9_3B2 with 350fmol pepmix_120min_3.5ul injection_8351_9_3B2 | 39 | 9 | 8351 |
| 3C6 | GBM | MES | unknown | wt | 48_UCSFslide8_3C6 with 350fmol pepmix_120min_3.5ul injection_8257_8_3C6 | 40 | 8 | 08257 |
| 3B3 | GBM | PRO | unknown | mutant | 49_UCSFslide9_3B3 with 350fmol pepmix_120min_3.5ul injection_8354_9_3B3 | 41 | 9 | 8354 |
| 3D5 | GBM | PRO | unknown | wt | 50_UCSFslide8_3D5 with 350fmol pepmix_120min_3.5ul injection_8345_8_3D5 | 42 | 8 | 08345 |
| 2B3 | GBM | PRO | unamplified | wt | 51_UCSFslide10_2B3 with 350fmol pepmix_120min_3.5ul injection_9259_10_2B3 | 43 | 10 | 9259 |

**Supplemental Table S2.** Lawrence codes for Chondroitin sulfate (CS) and heparan sulfate (HS) disaccharides. HexA= hexuronic acid, GalNAc= N-acetyl galactosamine, GlcNAc= N-acetyl glucosamine. S= sulfation position.


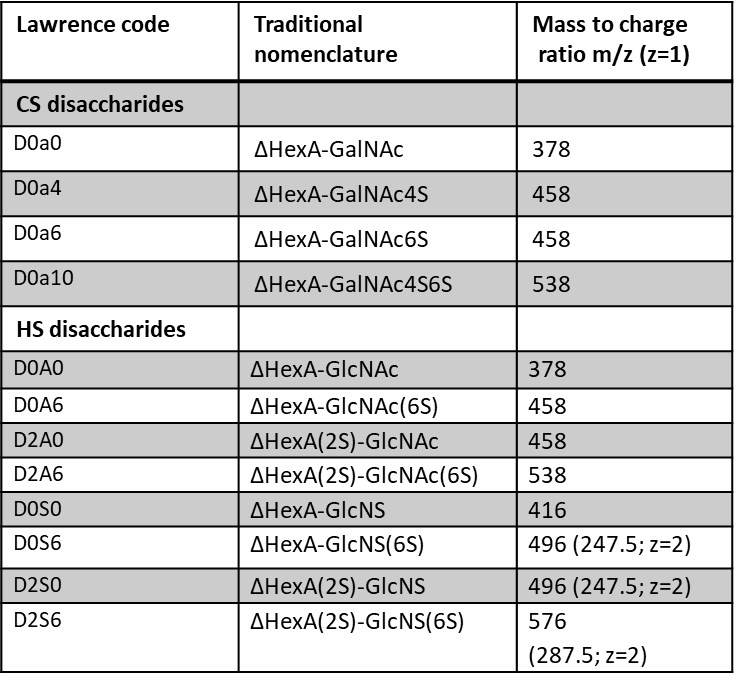


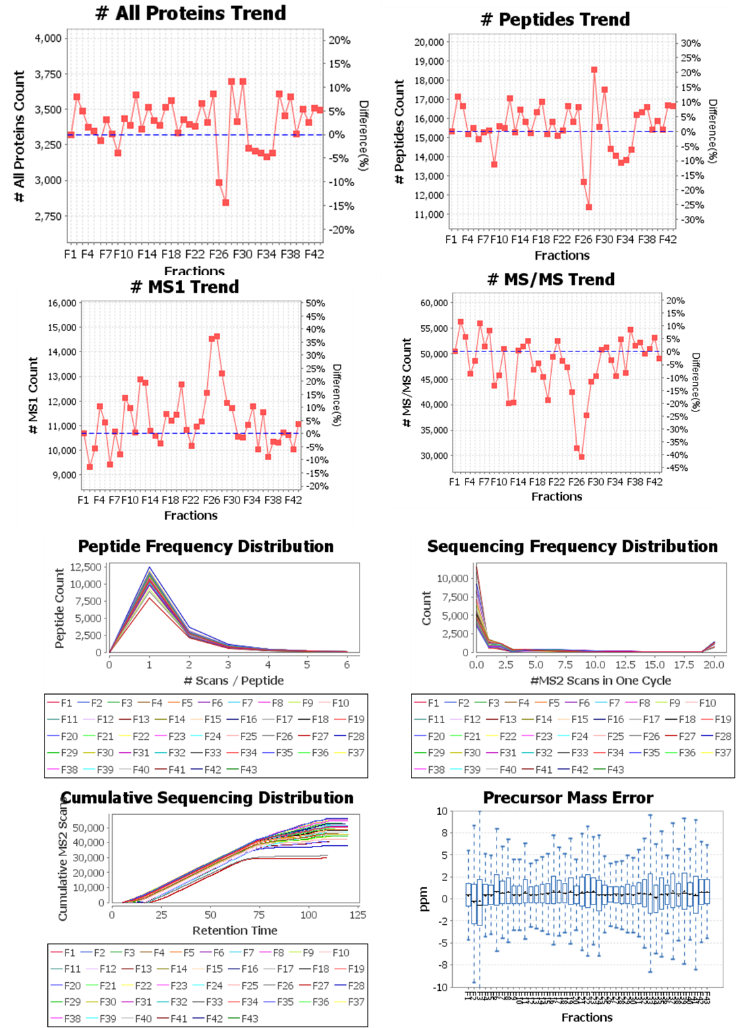


Supplemental Figure S1

A.

Supplemental Figure S1

B.


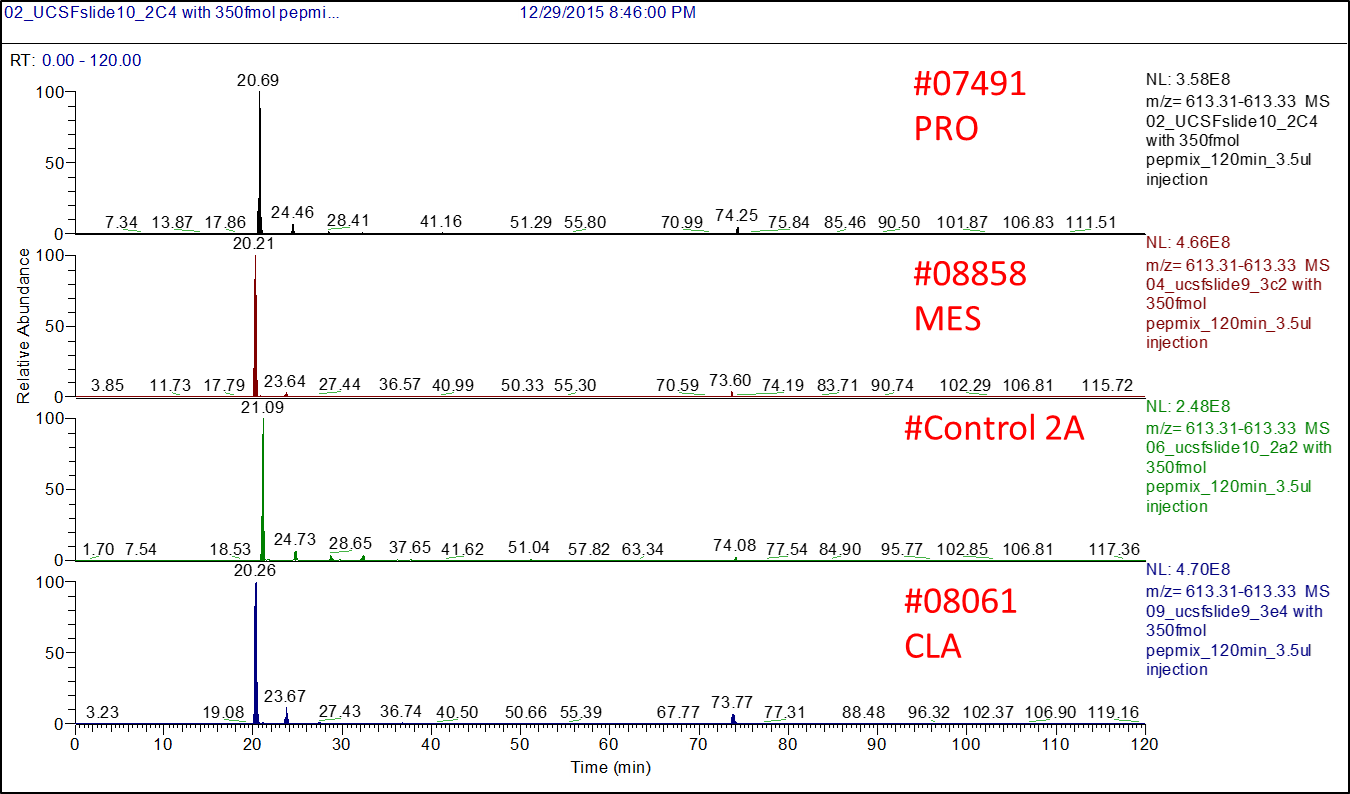


Supplemental Figure S1

C.

\\\\\\\\

#07491

PRO

#Control 2A

#08858

MES

#08061

CLA

Supplemental Figure S1


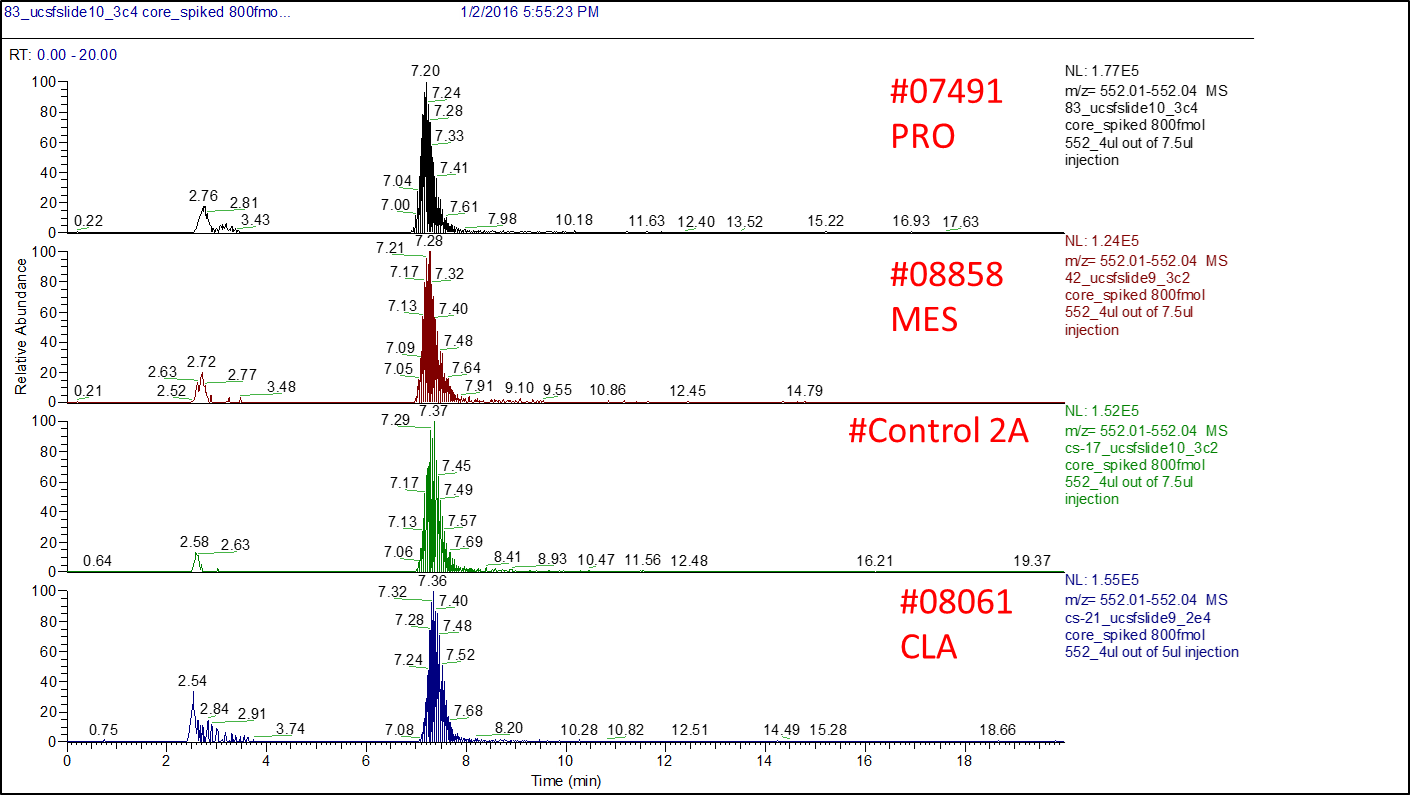


D.

**Supplemental Figure S1. A,** PEAKS Quality Control (QC) analysis. **B.** Representative extracted ion chromatogram (EIC) for *m/z* 613.32 peptide from spiked pierce retention time calibration mixture (350 fmol) for proteomic analysis. **C,** Representative extracted ion chromatogram (EIC) for spiked disaccharide internal standard m/z 552 (100 fmol) for HS glycomic analysis. **D,** Representative extracted ion chromatogram (EIC) for spiked disaccharide internal standard m/z 552 (800 fmol) for CS glycomic analysis.


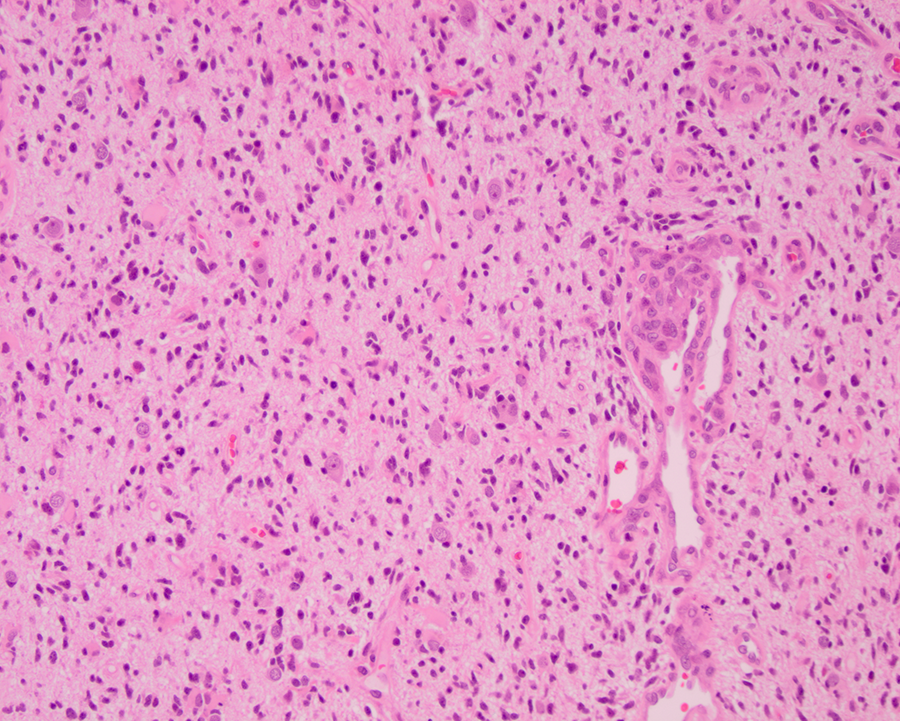

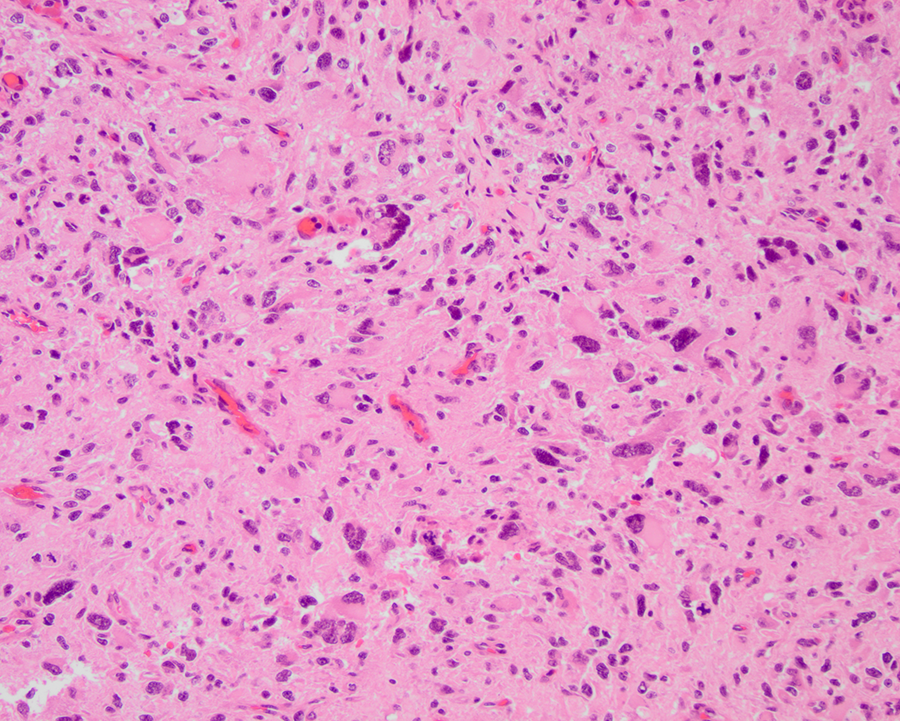


A

B

*

*

**Supplemental Figure S2.** Representative images of H&E-stained sections of TMA cores from (A) 08257 and (B) 08306 highlight tumor cell density in regions of the central tumor. Regions included in each core include tumor cells (arrow), vasculature (arrowhead), and eosinophilic regions containing both cell processes and extracellular matrix (asterisk). Line denotes 50 µm.

Supplemental Figure S3


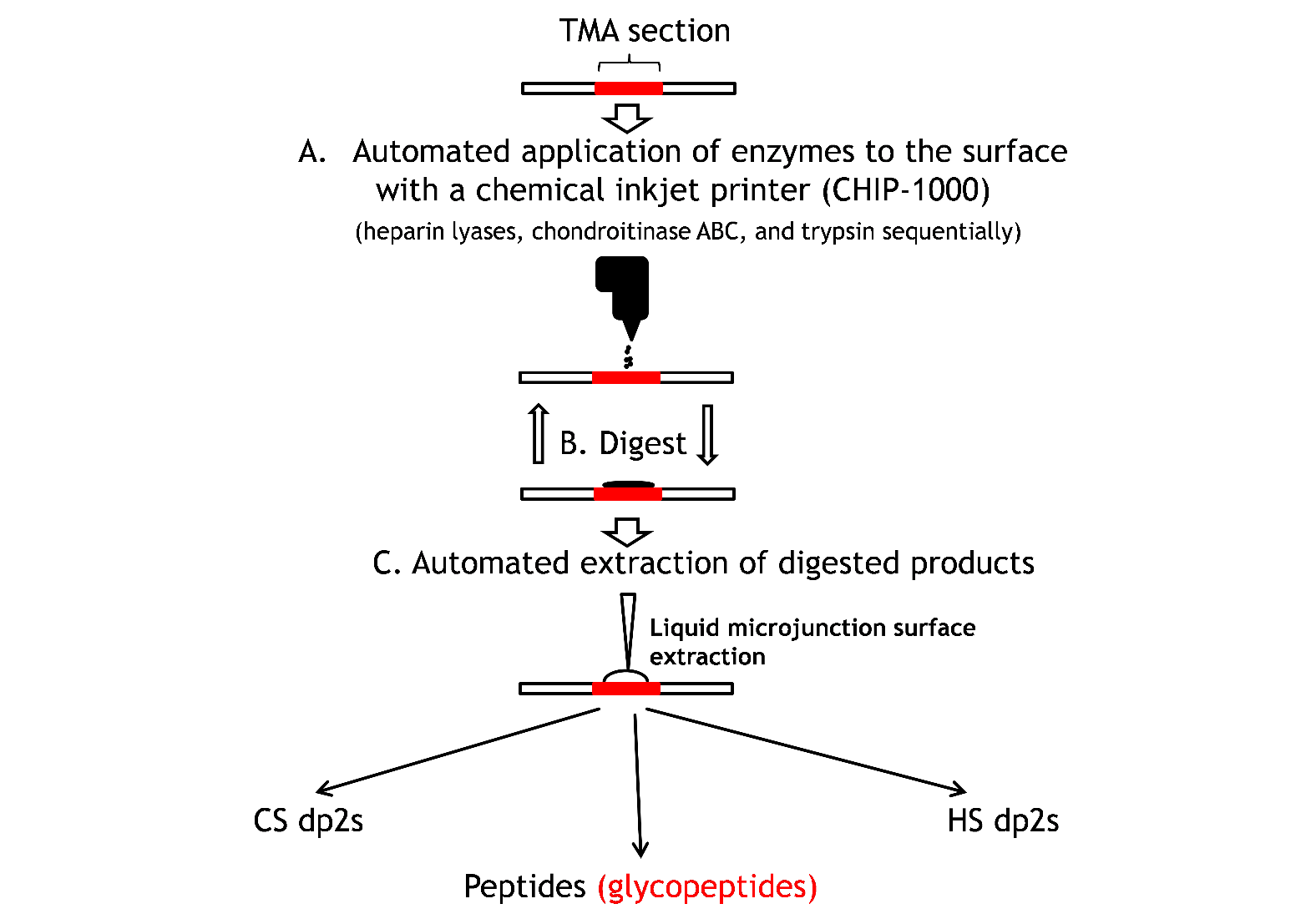


**Supplemental Figure S3.** Workflow for digestion of TMA slides using enzymes.


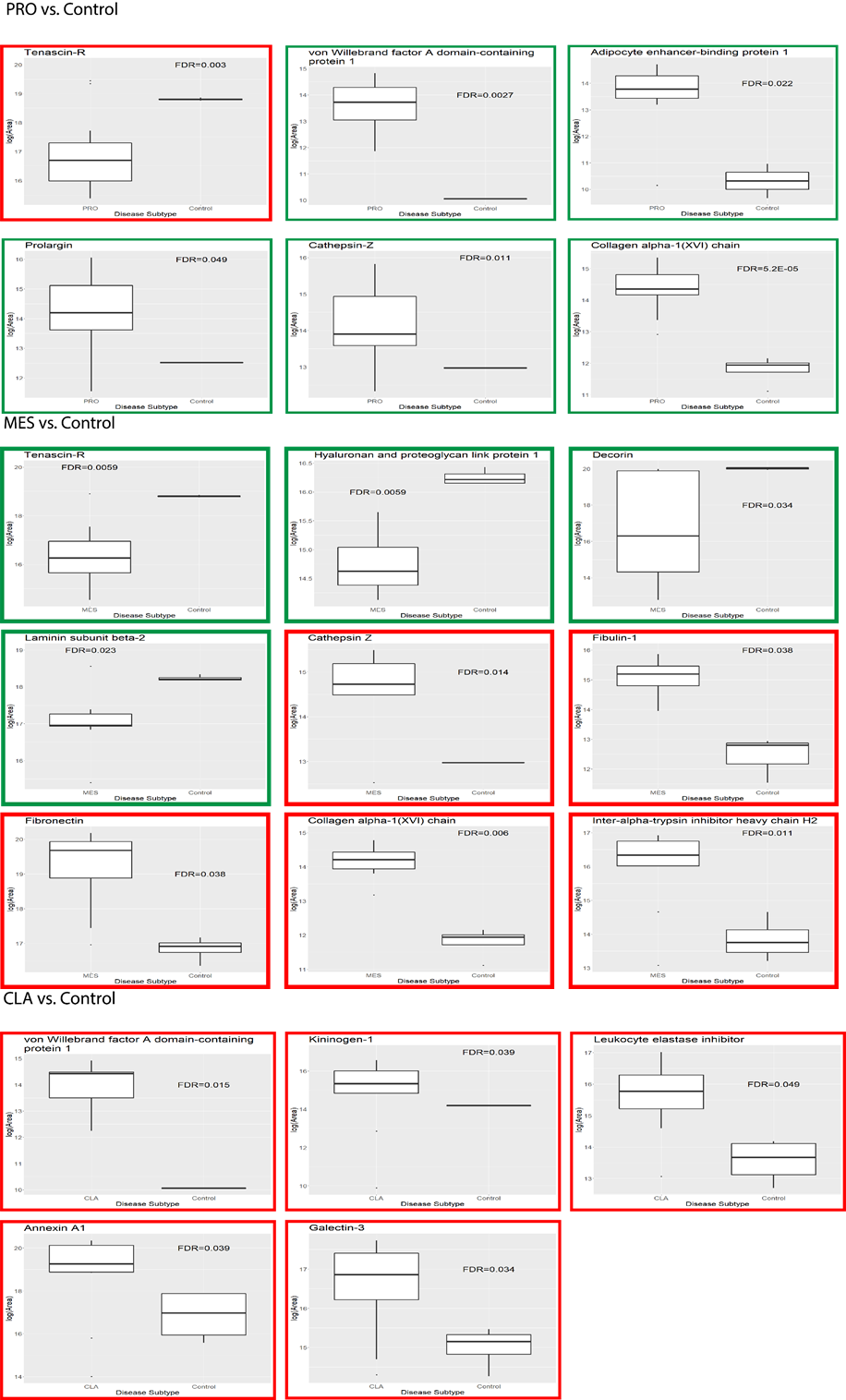
Supplemental Figure S4.

**A.**

Supplemental Figure S4

**B.**


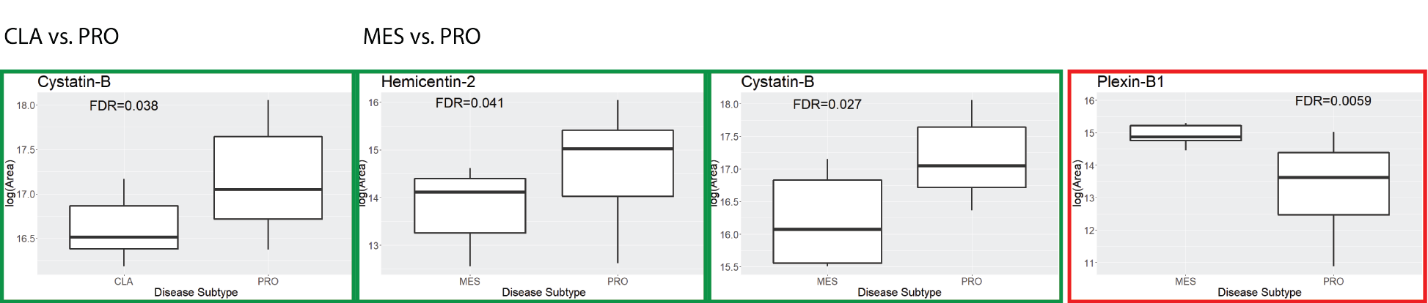


**Supplemental Figure S4.** Box plots representing normalized abundances for the top 5 increased-(*red box*) and decreased *(green box)* proteins (FDR < 0.05) from **A.** GBM subtypes vs. Control, **B.** GBM subtype pairwise comparisons. The comparisons between normalized log-transformed abundances were corrected for multiple comparisons.

Supplemental Figure S5.

**A.**


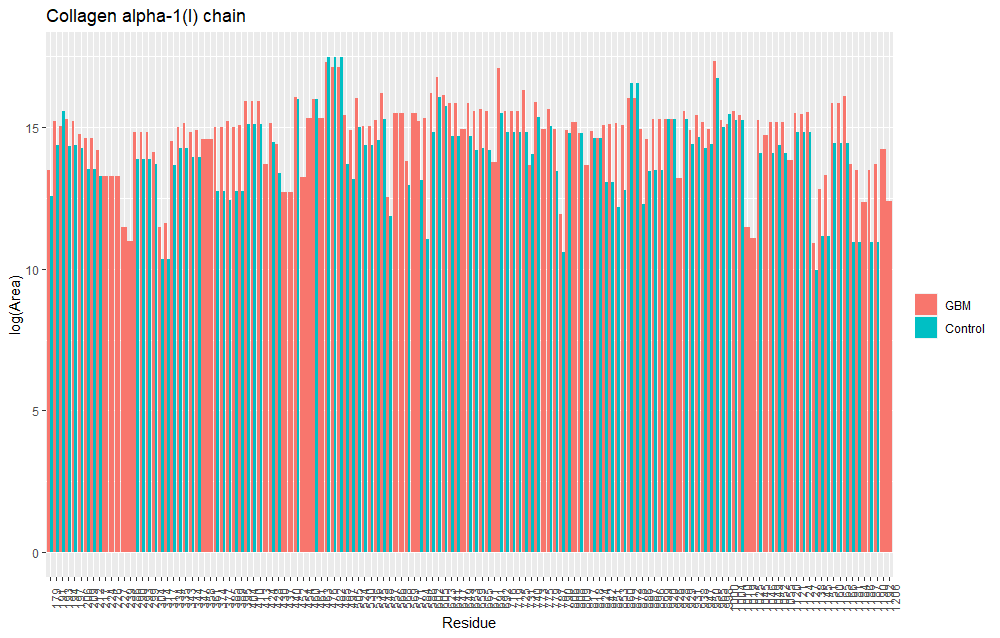


Supplemental Figure S5.

**B.**


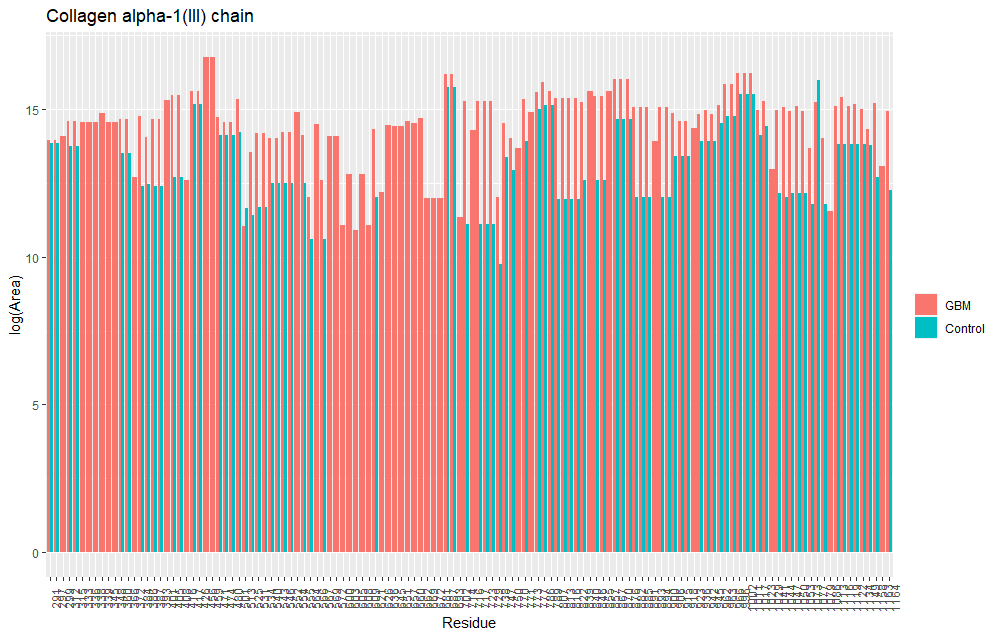


Supplemental Figure S5.


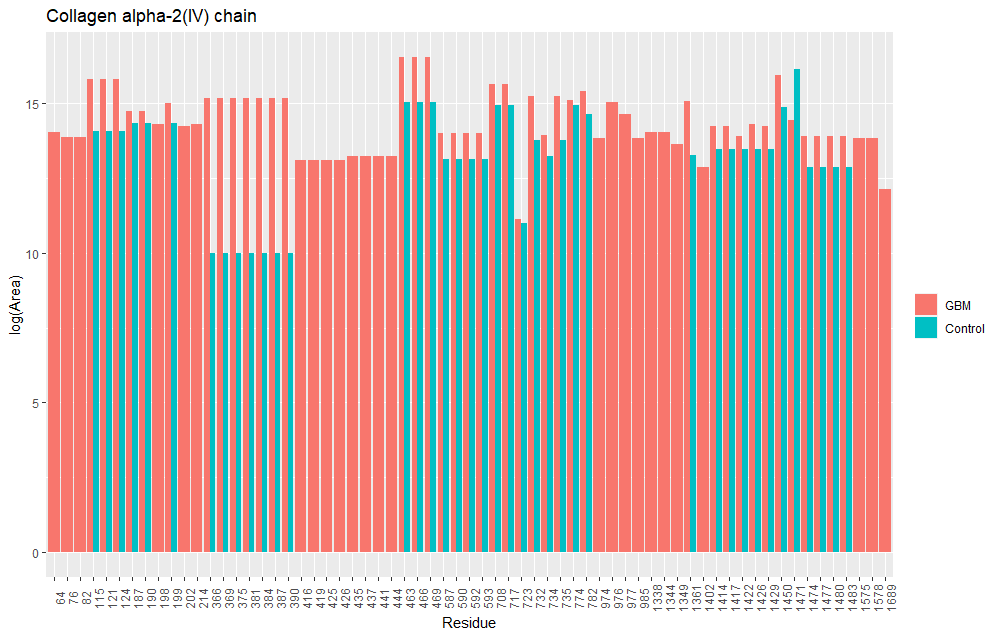


**C.**

**Supplemental Figure S5.** Bar plots representing log-transformed normalized abundances of hydroxyprolinated peptide residues for top significantly altered hydroxyprolinated collagen proteins for GBM vs. Control from Figure 3 and Supplemental File S12: A, CO1A1, B, CO1A3, C, CO4A2. The normalized log-transformed abundances were corrected for multiple comparisons.

Supplemental Figure S6.

**A.**


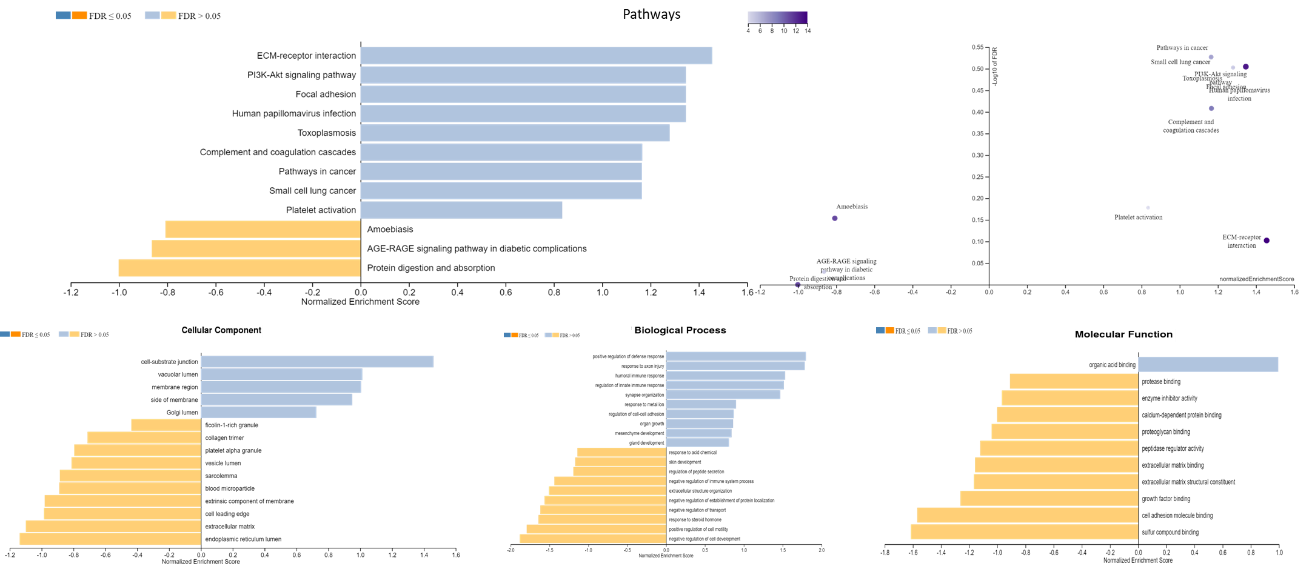


Supplemental Figure S6.

**B.**

Supplemental Figure S6.

**C.**

Supplemental Figure S6.

**D.**

Supplemental Figure S6.

**E.**

**Supplemental Figure S6. A.** Gene set enrichment analysis (GSEA) using Webgestalt. Pathways and gene ontology (GO): biological processes, cellular components, molecular functions. **B.** Network topology-based analysis (NTA) using TCGA RNAseq GBM database**. C.**  Extracellular matrix organization. **D.** Tumor development**. E.** Cell adhesion.

Supplemental Figure S7


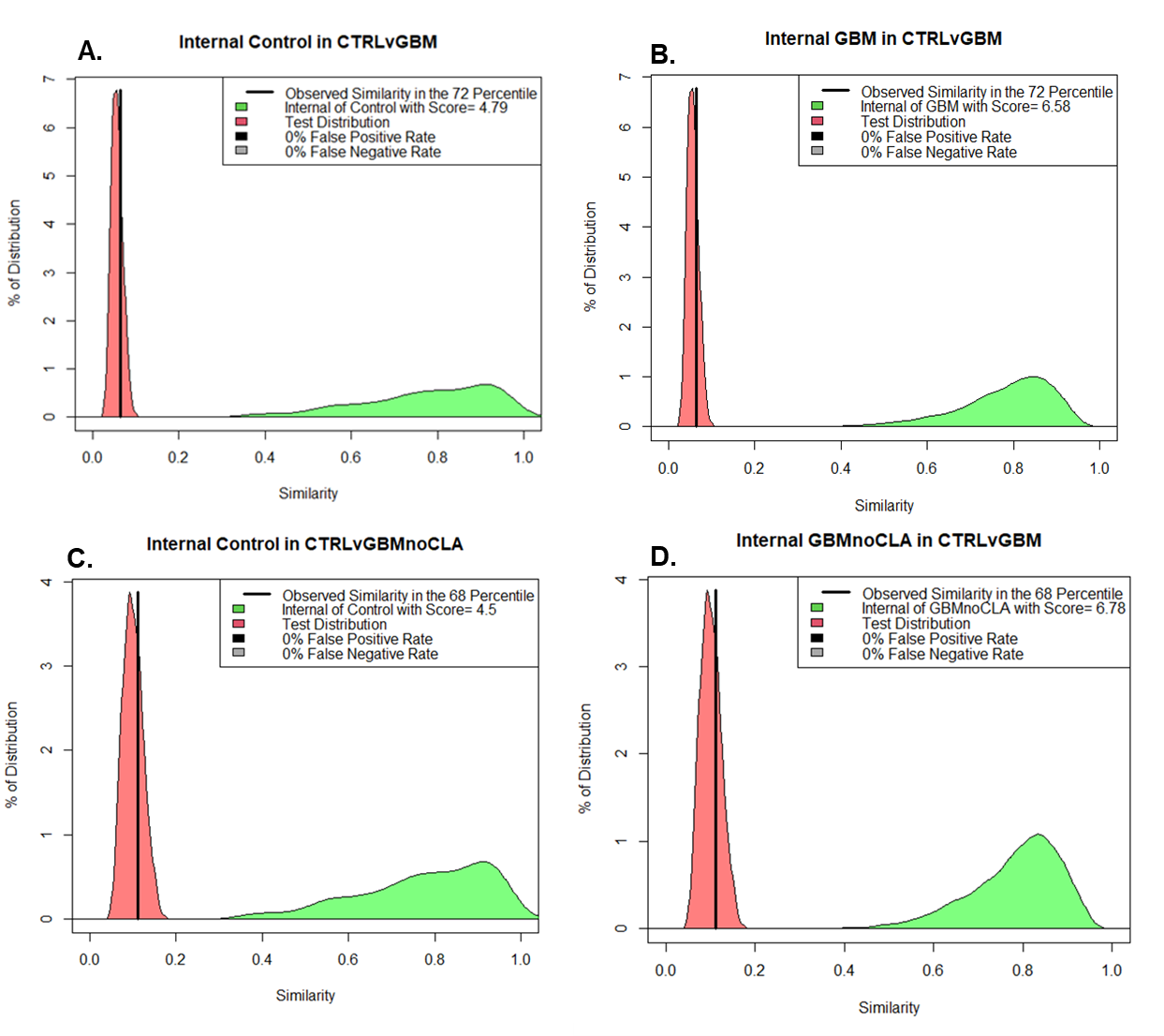


**Supplemental Figure S7.** Internal similarity distribution of GBM vs. Control (A, B) and GBM vs. Control after removing CLA subtype (C, D). For Internal Similarity distributions, broader distributions indicate a low degree of consistency within the sample, likely indicating a data quality or sample definition error. Narrowly defined Internal Similarities are indicative of reproducible results and are more likely to produce sufficient quality data for use in a comparison. The Internal Confidence Score is a punitively weighted z-score and represents the validity of usage of a sample group in a particular comparison; the same sample group data can be of high enough quality in one comparison only for it to fall below the quality threshold of 2 in another due to a higher or broader Test Similarity Distribution

Supplemental Figure S8


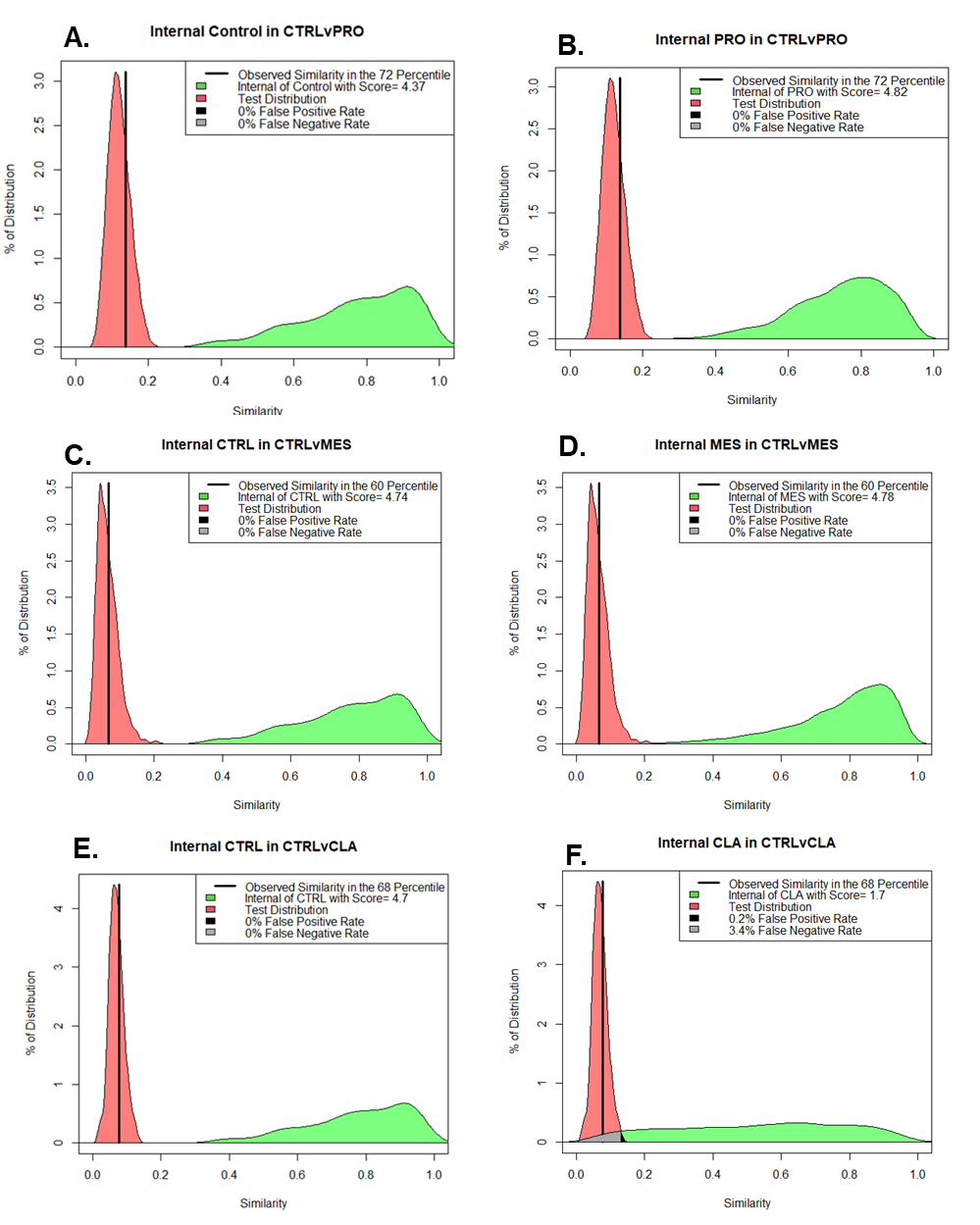


**Supplemental Figure S8.** Internal similarity distribution of Control vs. GBM subtypes viz. PRO (proneural), MES (mesenchymal), and CLA (classical). For Internal Similarity distributions, broader distributions indicate a low degree of consistency within the sample, likely indicating a data quality or sample definition error. Narrowly defined Internal Similarities are indicative of reproducible results and are more likely to produce sufficient quality data for use in a comparison. The Internal Confidence Score is a punitively weighted z-score and represents the validity of usage of a sample group in a particular comparison; the same sample group data can be of high enough quality in one comparison only for it to fall below the quality threshold of 2 in another due to a higher or broader Test Similarity Distribution.

Supplemental Figure S9


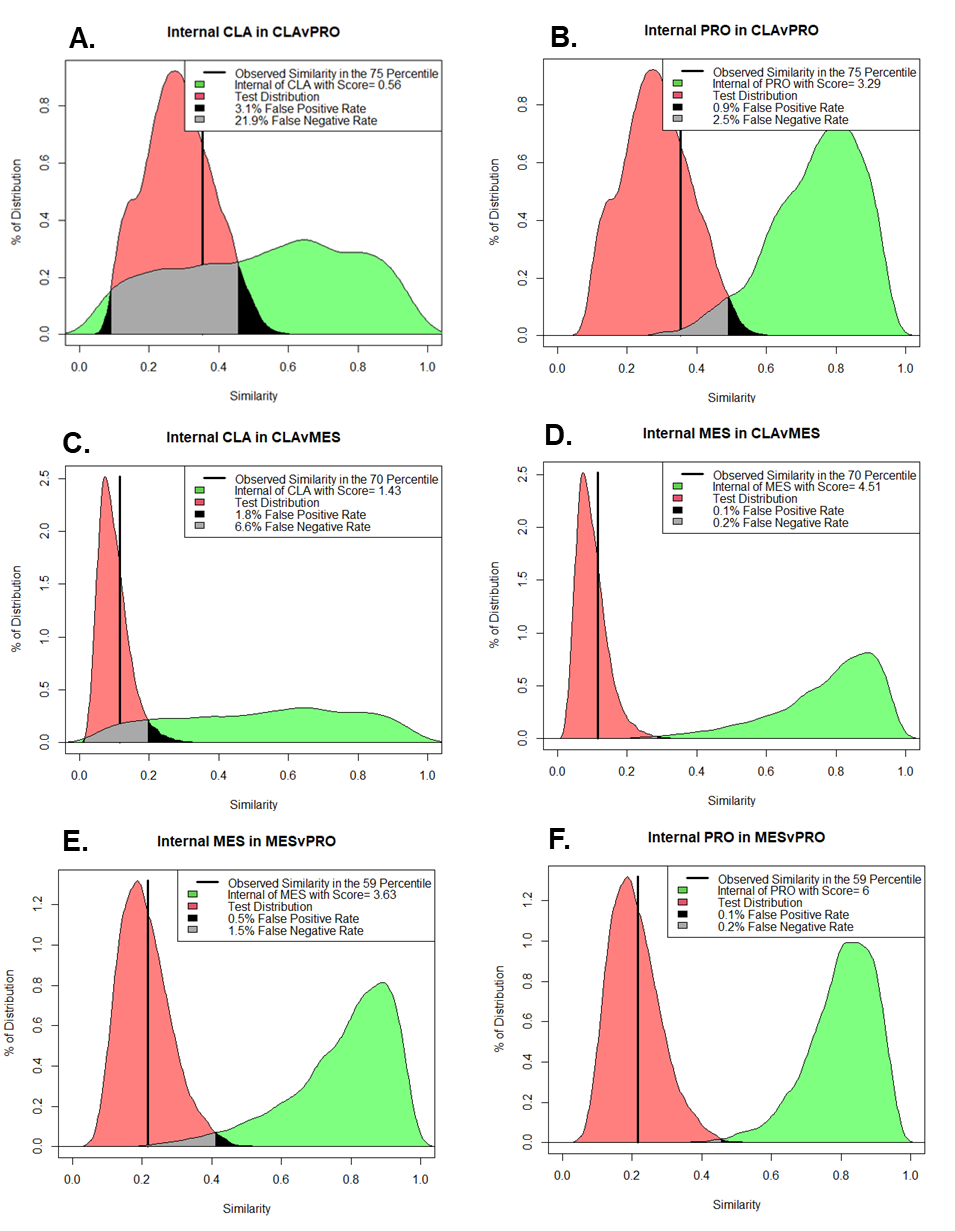


**Supplemental Figure S9.** Internal similarity distribution of subtypes comparisons CLA vs. PRO, CLA vs. MES, and MES vs. PRO. For Internal Similarity distributions, broader distributions indicate a low degree of consistency within the sample, likely indicating a data quality or sample definition error. Narrowly defined Internal Similarities are indicative of reproducible results and are more likely to produce sufficient quality data for use in a comparison. The Internal Confidence Score is a punitively weighted z-score and represents the validity of usage of a sample group in a particular comparison; the same sample group data can be of high enough quality in one comparison only for it to fall below the quality threshold of 2 in another due to a higher or broader Test Similarity Distribution.
